# Metabolic derangements are associated with impaired glucose delivery following traumatic brain injury

**DOI:** 10.1093/brain/awab255

**Published:** 2021-07-08

**Authors:** Jeroen Hermanides, Young T Hong, Monica Trivedi, Joanne Outtrim, Franklin Aigbirhio, Peter J Nestor, Matthew Guilfoyle, Stefan Winzeck, Virginia F J Newcombe, Tilak Das, Marta M Correia, Keri L H Carpenter, Peter J A Hutchinson, Arun K Gupta, Tim D Fryer, John D Pickard, David K Menon, Jonathan P Coles

**Affiliations:** 1 University Division of Anaesthesia, University of Cambridge, Cambridge, UK; 2 Wolfson Brain Imaging Centre, Department of Clinical Neurosciences, University of Cambridge, Cambridge, UK; 3 Queensland Brain Institute, University of Queensland, Brisbane, Australia; 4 Division of Neurosurgery, Department of Clinical Neurosciences, University of Cambridge, Cambridge, UK; 5 BioMedIA Group, Department of Computing, Imperial College, London, UK; 6 Department of Radiology, Addenbrooke’s Hospital, Cambridge, UK; 7 MRC Cognition and Brain Sciences Unit, University of Cambridge, Cambridge, UK

**Keywords:** traumatic brain injury, PET, cerebral blood flow, glucose metabolism, microdialysis

## Abstract

Metabolic derangements following traumatic brain injury are poorly characterized. In this single-centre observational cohort study we combined ^18^F-FDG and multi-tracer oxygen-15 PET to comprehensively characterize the extent and spatial pattern of metabolic derangements.

Twenty-six patients requiring sedation and ventilation with intracranial pressure monitoring following head injury within a Neurosciences Critical Care Unit, and 47 healthy volunteers were recruited. Eighteen volunteers were excluded for age over 60 years (*n* = 11), movement-related artefact (*n* = 3) or physiological instability during imaging (*n* = 4). We measured cerebral blood flow, blood volume, oxygen extraction fraction, and ^18^F-FDG transport into the brain (K_1_) and its phosphorylation (k_3_). We calculated oxygen metabolism, ^18^F-FDG influx rate constant (K_i_), glucose metabolism and the oxygen/glucose metabolic ratio. Lesion core, penumbra and peri-penumbra, and normal-appearing brain, ischaemic brain volume and k_3_ hotspot regions were compared with plasma and microdialysis glucose in patients.

Twenty-six head injury patients, median age 40 years (22 male, four female) underwent 34 combined ^18^F-FDG and oxygen-15 PET at early, intermediate, and late time points (within 24 h, Days 2–5, and Days 6–12 post-injury; *n* = 12, 8, and 14, respectively), and were compared with 20 volunteers, median age 43 years (15 male, five female) who underwent oxygen-15, and nine volunteers, median age 56 years (three male, six female) who underwent ^18^F-FDG PET.

Higher plasma glucose was associated with higher microdialysate glucose. Blood flow and K_1_ were decreased in the vicinity of lesions, and closely related when blood flow was <25 ml/100 ml/min. Within normal-appearing brain, K_1_ was maintained despite lower blood flow than volunteers. Glucose utilization was globally reduced in comparison with volunteers (*P* < 0.001). k_3_ was variable; highest within lesions with some patients showing increases with blood flow <25 ml/100 ml/min, but falling steeply with blood flow lower than 12 ml/100 ml/min. k_3_ hotspots were found distant from lesions, with k_3_ increases associated with lower plasma glucose (Rho −0.33, *P* < 0.001) and microdialysis glucose (Rho −0.73, *P* = 0.02). k_3_ hotspots showed similar K_1_ and glucose metabolism to volunteers despite lower blood flow and oxygen metabolism (*P* < 0.001, both comparisons); oxygen extraction fraction increases consistent with ischaemia were uncommon.

We show that glucose delivery was dependent on plasma glucose and cerebral blood flow. Overall glucose utilization was low, but regional increases were associated with reductions in glucose availability, blood flow and oxygen metabolism in the absence of ischaemia. Clinical management should optimize blood flow and glucose delivery and could explore the use of alternative energy substrates.

## Teaser

Hermanides *et al*. show that following TBI, glucose delivery is limited by plasma glucose and cerebral blood flow. Overall glucose utilization is low, but regional increases are associated with decreases in glucose availability, blood flow and oxygen metabolism in the absence of ischaemia.

## Introduction

Acute derangements in cerebral glucose metabolism (CMRG) are common following traumatic brain injury (TBI).^1-4^ These have been attributed to anaerobic glycolysis in the presence of classical ischaemia, but other pathophysiological mechanisms may also be responsible.^5–7^ Microdialysis shows marked reductions in brain glucose^8^ with modest decreases in plasma glucose within the normal range resulting in metabolic crisis^9^ and poor outcome.^10^ In addition to altered relationships between plasma and brain glucose,^8^ substantial spatial and temporal heterogeneity in glucose utilization exists,^2^ with increases in non-oxidative glucose utilization seen across white matter,^11^ and in relation to brain lesions.^2^^,^^12^ The changes in brain glucose metabolism represent complex interactions between glucose availability (plasma glucose), delivery (blood flow), uptake across the blood–brain barrier (transport), entry into the glycolytic pathway (phosphorylation), and oxidative metabolism of glucose (characterized as the metabolic ratio of oxygen-to-glucose utilization). Addressing these complex inter-relationships, while accounting for spatial variations in physiology, requires appropriate tools.

PET using the glucose analogue ^18^F-fluorodeoxyglucose (^18^F-FDG) and multi-tracer oxygen-15 ($H_{2}^{15}$O, ^15^O_2_ and C^15^O) allow imaging of regional CMRG and kinetic parameters,^13–15^ and comparison with cerebral blood flow (CBF), oxygen metabolism (CMRO_2_) and oxygen extraction fraction (OEF). Hattori *et al*.^2^ showed that ^18^F-FDG transport into brain, expressed by the rate constant K_1_, was reduced within the vicinity of contusions following TBI. Glucose uptake depends on supply (plasma glucose and CBF) with transport facilitated via GLUT-1.^16^ Reductions within the vicinity of lesions could be explained by a decrease in glucose availability, delivery, transport capacity, or a combination of these factors. Increases in glucose phosphorylation have also been seen following TBI,^2^^,^^12^ and tend to occur on the background of an overall reduction in phosphorylation activity (measured by the ^18^F-FDG rate constant k_3_).^11^

An integrated understanding of these acute metabolic derangements following clinical TBI is critical for development of treatments that improve outcome. Using a combination of ^18^F-FDG and ^15^O PET, with brain microdialysis in a subset of patients, we investigate how derangements in ^18^F-FDG kinetic parameters relate to visible TBI lesions, and changes in plasma and microdialysis glucose, CBF, CMRO_2_, and OEF increases consistent with classical ischaemia.

## Materials and methods

In this single-centre observational cohort study, 26 TBI patients requiring intracranial pressure (ICP) monitoring within intensive care and 47 healthy volunteers underwent ^18^F-FDG and ^15^O PET between February 1998 and July 2014. We excluded volunteers aged >60 years, technically inadequate imaging studies and those with large variations of arterial pressure or PaCO_2_. Some data have been reported previously,^6^^,^^17^ but none for the aims of the current manuscript.

Studies were conducted in accordance with 1964 Declaration of Helsinki and later amendments following approval by Cambridgeshire Research Ethics Committee (REC 97/290) and UK Administration of Radioactive Substances Advisory Committee. Volunteers provided informed consent, and assent obtained from patient representatives with consent at follow-up if capacity was regained.

### Clinical protocols

#### Patients

Protocolized and graded interventions used for cerebral perfusion pressure (CPP) and ICP control^18^ included sedation (propofol 3–5 mg/kg/h and fentanyl 1–4 µg/kg/h) and neuromuscular blockade, surgery for space-occupying lesions, drainage of CSF, vasoactive agents for CPP increment, osmotic therapy, mild hyperventilation [aimed at ∼4.5 kPa (35 mmHg)], mild-to-moderate hypothermia (33–36°C), decompressive craniectomy, and barbiturate metabolic depression. Management aimed for ICP < 20 mmHg and CPP > 65 mmHg^19^ with tight control of physiology during PET. Outcome was recorded using the Glasgow Outcome Score (GOS)^20^ at 6 months post-TBI.

#### Healthy volunteers

Blood pressure, pulse oximetry, and arterial blood gases were monitored to ensure physiological stability during imaging.

### Imaging

PET data were acquired on a GE Advance PET scanner (GE Medical Systems). Parametric maps of CBF, cerebral blood volume (CBV), OEF, CMRO_2_ and ^18^F-FDG transport into the brain (K_1_), phosphorylation (k_3_), and influx rate constant (K_i_) (Supplementary Fig. 1), together with CMRG were calculated at the voxel and regional level as previously described.^21–23^ A more detailed description is provided within the Supplementary material. We categorized PET studies into those performed within 24 h (early), between 2–5 days (intermediate), and 6–12 days (late) following TBI. Parametric maps were co-registered to acute anatomy using structural X-ray CT. In addition, and where available, MRI obtained at follow up were co-registered to early CT.

### Microdialysis

Microdialysis catheters (CMA70 10-mm membrane, CMA) were inserted into the cerebral parenchyma as part of standard clinical monitoring. Vials were collected every 20 min and glucose, glutamate, lactate and pyruvate were determined using the CMA600 bedside microdialysis analyser.^24^ We calculated the mean microdialysis and plasma glucose during ^18^F-FDG PET.

### Imaging analysis

Imaging data were analysed using a combination of custom-designed automated software (PETAn)^25^ and several software packages, including Statistical Parametric Mapping (SPM, Wellcome Department of Imaging Neuroscience, University College London), MATLAB (MathWorks, Inc), Analyze (AnalyzeDirect, Inc) and Advanced Normalization Tools (ANTs).^26^ Structural data (CT or T_1_-weighted MRI) were edited to extract a mask that identified brain tissue voxels and excluded extracranial tissue, CSF, and extra-axial haematomas. All whole brain and region of interest data were masked prior to calculation of PET parameters. In healthy volunteers a whole brain region was used incorporating mixed grey and white matter. In patients, T_1_-weighted MRI obtained at follow-up were non-rigidly registered with acute CT using ANTs.

Structural T_1_-weighted MRI acquired in healthy volunteers, and at follow-up in patients, were processed on a specific TBI pipeline^27^ incorporating segmentation into grey and white matter regions of interest. These regions of interest were applied to the Jacobian determinant images^28^ providing the amount of contraction or expansion required to normalize the subjects brain into template space (Supplementary material).

#### Structural regions of interest

**Figure 1:**
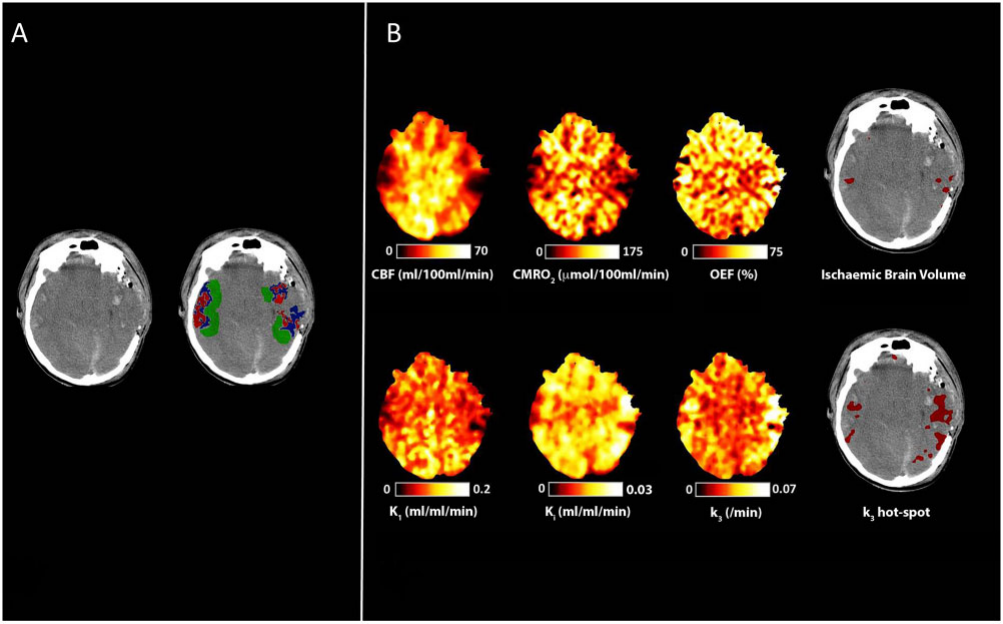
**Peri-lesional hyperglycolysis in the absence of cerebral ischaemia.** CT, CBF, CMRO_2_, OEF, and ^18^F-FDG kinetic parameters obtained in a 29-year-old male within 24 h of severe TBI following a road traffic accident. Plasma glucose during imaging was 6.6 mmol/l. (**A**) CT demonstrates bilateral temporal and parietal haemorrhagic contusions. Regions of interest are defined for haemorrhagic lesion (core, red), hypodense tissue (penumbra, blue), and a 1 cm border zone of normal-appearing tissue (peri-penumbra, green). (**B**) Co-registered parametric maps of CBF, CMRO_2_, OEF, K_1_, K_i_ and k_3_. The regions with a critical increase in OEF above the individually calculated threshold (ischaemic brain volume) and region of increased phosphorylation activity (k_3_ hotspot) are both shown in red overlying the CT scan. The ischaemic brain and k_3_ hotspot volumes were 7 ml and 56 ml, respectively, and overlap between these two tissue classes in this patient was 0.4 ml.

Lesion-based regions of interest were drawn on CT using a bespoke segmentation tool (Imseg V1.8, BioMedIA, London, UK). We defined haemorrhagic contusion core and low signal intensity penumbra (Fig. 1A). In addition, we drew a 1 cm cuff of normal-appearing tissue adjacent to penumbra (peri-penumbra). When present, we drew a 10 mm circular cuff of tissue centred on the microdialysis catheter tip. All other normal-appearing tissue was defined as the ‘normal-appearing’ region of interest.

#### Physiological regions of interest

We used the mean and standard deviation (SD) of k_3_ voxels within normal-appearing brain of TBI patients to identify regional hotspots. These k_3_ hotspot regions of interest were defined as the region where k_3_ voxels were found above the upper 95% confidence interval threshold calculated for each individual patient using the mean plus 1.96 SDs of normal-appearing brain voxels.

We estimated an individualized critical OEF threshold using a previously validated technique to define and calculate the ischaemic brain volume (IBV).^17^ A more detailed description is provided in the Supplementary material. We examined the spatial location, underlying physiology and mismatch between k_3_ hotspots and the IBV. MRI obtained at follow-up were co-registered to acute CT. The k_3_ hotspot regions of interest (minus lesion core and penumbra) were superimposed to explore possible relationships between increased k_3_ and tissue fate.

### Statistical analyses

Data were analysed using R (R Foundation for Statistical Computing, Vienna, Austria), and expressed as median (interquartile range, IQR) unless otherwise stated. Correlations were calculated using linear regression and, in the case of small datasets, Spearman rank. For comparisons between groups, we used the Mann-Whitney U-test and Kruskal-Wallis with Dunn’s test for *post hoc* comparisons. The Dice similarity coefficient was used to determine the degree of spatial overlap between k_3_ hotspots and the IBV.^29^ Locally weighted scatterplot smoothing (LOWESS)^30^ was used to plot relationships using whole brain voxel data using a 2.5% weighted sample. Results were interpreted taking into consideration that a Bonferroni correction for use of 11 variables [K_1_, k_2_, k_3_, K_i_, CMRG, lumped constant, CBF, CMRO_2_, OEF, CBV, and CMRO_2_/CMRG metabolic ratio (MR)] would require *P* < 0.005 for formal statistical significance.

### Data availability

The data that support the findings of this study are available from the corresponding author, upon reasonable request.

## Results

Additional data are presented within the Supplementary material.

### Study population

We analysed data from 20 healthy volunteers following ^15^O PET and nine healthy volunteers following ^18^F-FDG PET, with no significant neurological/psychiatric illness, and 34 combined PET sessions (^18^F-FDG and ^15^O) in 26 TBI patients with stable ventilation and CPP as per protocolized clinical management. Eleven healthy volunteers were excluded for age >60 years, three for movement-related artefact and four with physiological instability during imaging (variations in PaCO_2_ > 0.5 kPa).

Patients had severe TBI with a Glasgow Coma Score (GCS) ≤ 8 or presented with moderate TBI (GCS 9–12), but deteriorated, requiring sedation and ventilation for management of ICP. Of the 34 PET sessions in patients, 12 were within 24 h (early), eight between 2–5 days (intermediate), and 14 between 6–12 days (late) after TBI. Eight subjects underwent imaging on two occasions, but for one of these only the CBF from the late session was analysed. Table 1 summarizes participant characteristics. The ^18^F-FDG healthy volunteers were older than the ^15^O healthy volunteers and TBI patients [56 (53–57) versus 43 (31–47) and 40 (26–52) years, respectively; both comparisons *P* < 0.01], while ^15^O healthy volunteers and TBI patients were similar in age (*P* = 0.95). Female participants were more common within ^18^F-FDG healthy volunteers compared to the ^15^O healthy volunteers and TBI patients [6 of 9 (67%) versus 5 of 20 (25%) and 4 of 26 (15%), respectively; both comparisons *P* < 0.05], while ^15^O healthy volunteers and TBI patients were similar in terms of gender (*P* = 0.47).

**Table 1:** Participant characteristics

	Healthy volunteers		TBI patients
	^18^F-FDG (*n* = 9)	^15^O (*n* = 20)	Total (*n* = 26)
Age, years	56 (53–57)	43 (31–47)	40 (26–52)
Gender, female/male	6/3	5/15	4/22
GCS post-resuscitation	–	–	6 (3–9)
Injury mechanism			
Fall	–	–	10
Road traffic collision	–	–	16
Marshall classification			
Diffuse injury I	–	–	0
Diffuse injury II	–	–	5
Diffuse injury III	–	–	0
Diffuse injury IV	–	–	0
Evacuated mass lesion V	–	–	13
Non-evacuated mass lesion VI	–	–	8
Primary lesion			
Subdural haematoma	–	–	5
Contusion	–	–	18
DAI, IVH and tSAH	–	–	3
Craniectomy	–	–	12
Barbiturate coma	–	–	2
Hypothermia	–	–	1
Blood glucose, mmol/l	4.6 (4.4–5.4)	–	5.8 (5.3–6.7)
Outcome (GOS)	–	–	3 (3–4)^a^

Characteristics of the TBI patients who underwent combined ^18^F-FDG and ^15^O PET, and the healthy volunteers who underwent separate ^18^F-FDG and ^15^O PET sessions. Data shown are median (IQR). DAI = diffuse axonal injury; GCS = Glasgow Coma Scale; IVH = intraventricular haemorrhage; tSAH = traumatic subarachnoid haemorrhage. Blood glucose is the mean value of this variable over the course of each PET study.

a Two missing data-points for GOS.

### Glucose availability, delivery and transport

#### Brain glucose is correlated with plasma glucose

Plasma glucose during PET was higher following TBI compared with healthy volunteers 5.8 (5.3–6.7) versus 4.6 (4.4–5.4) mmol/l (*P* < 0.001). Microdialysis data were available for 10 PET sessions from eight patients. On two occasions the microdialysis catheter was inserted within the vicinity of injured tissue. Plasma and microdialysis glucose in this subset ranged from 4.9–8.9 mmol/l and 0.4–4.1 mmol/l, respectively, and increases in plasma glucose were associated with higher microdialysis glucose (Rho = 0.64, *P* = 0.047; Supplementary Fig. 5). Brain tissue glucose concentration calculated using the ^18^F-FDG kinetic parameters was related to plasma glucose within healthy volunteers (R^2^ = 0.47, *P* = 0.04) and all TBI regions (R^2^ = 0.16, *P* < 0.001). In patients, this relationship was strongest within normal-appearing brain and peri-penumbra (R^2^ = 0.66, *P* < 0.001 and R^2^ = 0.69, *P* < 0.001, respectively), in comparison with core and penumbra (R^2^ = 0.06, *P* = 0.17 and R^2^ = 0.25, *P* < 0.01, respectively) (Supplementary Fig. 6).

#### Glucose delivery varies between normal-appearing, perilesional and lesion core tissue

**Figure 2:**
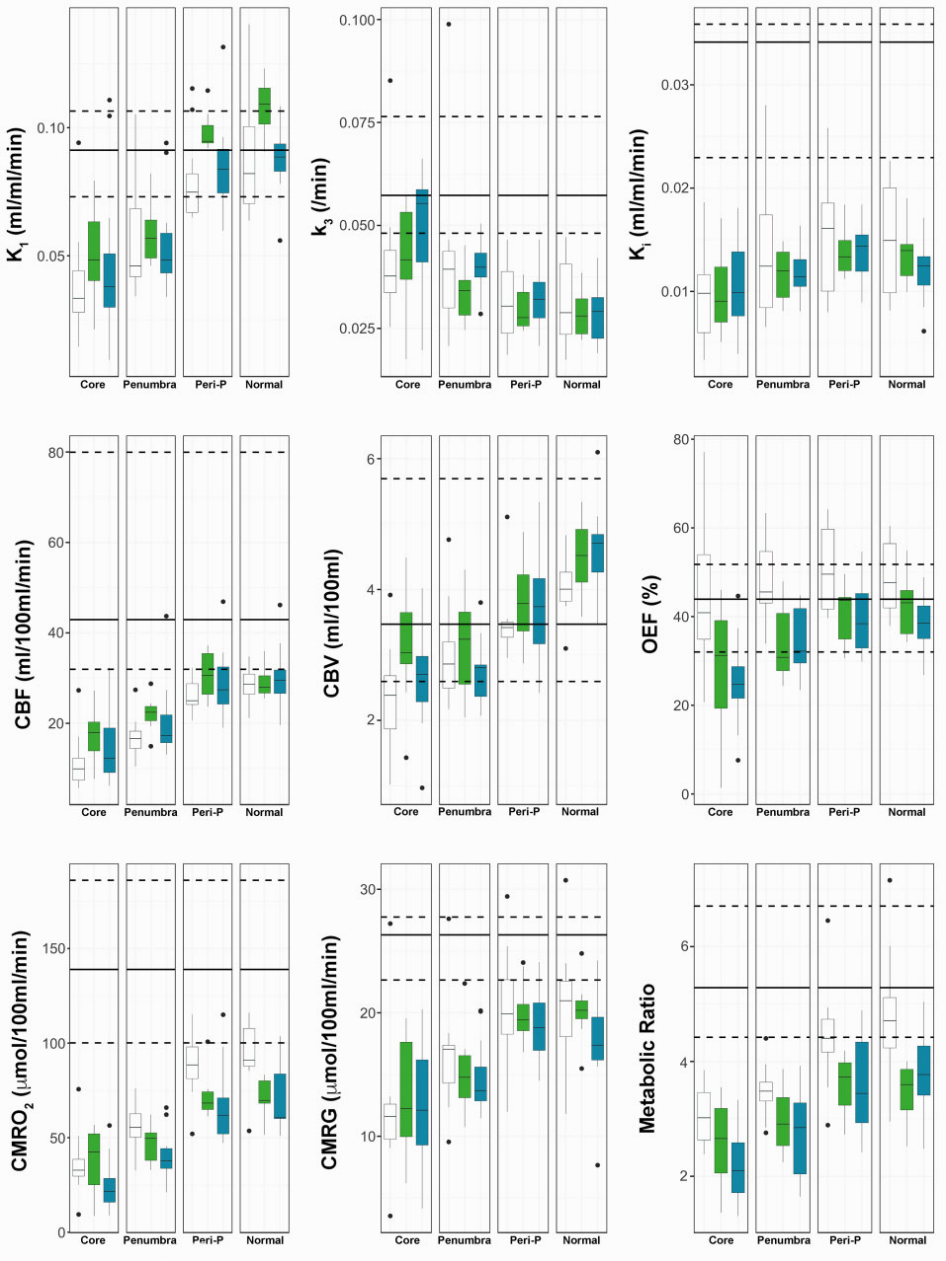
**Spatial and temporal pattern of metabolic parameters.** Box-and-whisker plots of the ^18^F-FDG kinetic parameters (K_1_, k_3_, K_i_), CBF, CBV, OEF, CMRO_2_, CMRG, and CMRO_2_/CMRG metabolic ratio for the different regions of interest in patients within 24 h (Early; white), Days 2–5 (Intermediate; green) and Days 6–12 (Late, blue) post-injury. The horizontal line within each box denotes the median value, the lower and upper boundaries the 25th and 75th centile, the vertical lines the 10th and 90th centile, and the closed circles outlying data-points. The solid and dashed black lines represent the median and the full range of values for healthy volunteers, respectively. For the metabolic ratio the median and range in healthy volunteers is calculated from two different cohorts of subjects.

CBF and K_1_ were decreased in core and penumbra as compared to peri-penumbra and normal-appearing brain in patients (*P* < 0.001; Table 2). When compared with healthy volunteers, normal-appearing brain in patients had lower CBF (*P* < 0.001), but similar K_1_. There were no significant changes in CBF and K_1_ within lesion and normal-appearing brain regions at different time points (Fig. 2 and Supplementary Table 1).

**Table 2:** Regional metabolic parameters

	Patients				Healthy volunteers		
	Core	Penumbra	Peri-penumbra	Normal appearing	*P*-value^a^		*P*-value^b^
K_1_, ml/ml/min	0.039 (0.021)^c,d^	0.049 (0.018)^c^	0.084 (0.022)^e^	0.091 (0.025)	<0.001	0.091 (0.007)	0.720
k_2_,/min	0.154 (0.069)	0.131 (0.048)^c^	0.156 (0.052)	0.173 (0.055)	<0.001	0.128 (0.029)	<0.001
k_3_,/min	0.044 (0.021)^c,d^	0.039 (0.010)^c^	0.030 (0.012)	0.029 (0.010)	<0.001	0.057 (0.009)	<0.001
K_i_, ml/ml/min	0.009 (0.006)^c,d^	0.012 (0.004)	0.014 (0.004)	0.013 (0.005)	<0.001	0.034 (0.008)	<0.001
CMRG, µmol/100 ml/min	11.9 (5.4)^c,d^	14.9 (4.3)^c^	19.5 (4.1)^e^	19.9 (4.9)	<0.001	26.3 (1.9)	<0.001
LC	0.48 (0.07)^c,d^	0.47 (0.05)^c^	0.43 (0.05)^e^	0.41 (0.04)	<0.001	0.56 (0.04)	<0.001
CBF, ml/100 ml/min	12.2 (10.7)^c,d^	18.1 (6.4)^c^	27.4 (8.1)^e^	28.6 (4.9)	<0.001	42.9 (11.5)	<0.001
CBV, ml/100 ml	2.7 (0.9)^c,d^	2.8 (0.8)^c^	3.5 (0.9)^e^	4.3 (0.9)	<0.001	3.5 (0.5)	<0.001
CMRO_2_, µmol/100 ml/min	29.6 (23.8)^c,d^	45.6 (20.6)^c^	71.9 (27.3)^e^	82.1 (30.5)	<0.001	139.0 (39)	<0.001
OEF, %	30.0 (20.1)^c,d^	38.5 (14.4)	42.3 (12.2)	42.1 (10.4)	<0.001	43.9 (4.5)	0.685
Metabolic ratio	2.6 (1.3)^c,d^	2.9 (0.8)^c^	4.0 (1.2)^e^	4.0 (1.2)	<0.001		

^18^F-FDG kinetic parameters, CMRG, lumped constant (LC), CBF, CBV, CMRO_2_, OEF and CMRO_2_/CMRG metabolic ratio for the different regions of interest in patients and healthy volunteers. Values are median (IQR).

a Kruskal-Wallis test for comparison between core, penumbra, peri-penumbra and normal-appearing regions of interest within patients, with subsequent *post hoc* Dunn’s tests surviving correction for multiple comparisons (*P* < 0.005) between each lesion region of interest and normal-appearing brain^c^, between core and peri-penumbra^d^, and between peri-penumbra and penumbra^e^.

b Mann-Whitney U-test for comparison between the normal-appearing region of interest in patients versus healthy volunteers.

#### Cerebral blood flow is an important determinant of brain glucose transport

**Figure 3:**
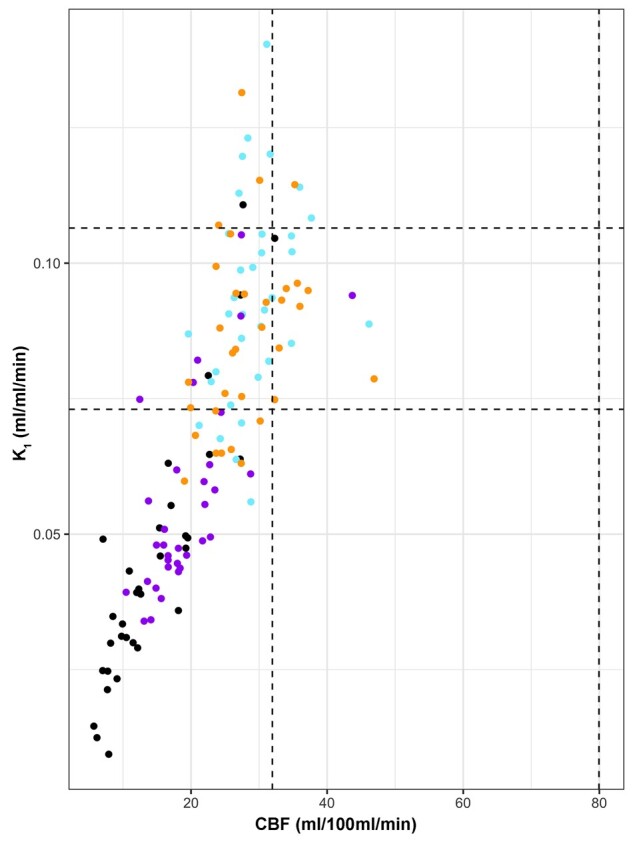
**Relationship between glucose transport and CBF based on region of interest data.** Scatterplot of ^18^F-FDG transport (K_1_) and CBF in patient regions of interest: lesion core (black), penumbra (purple), peri-penumbra (blue) and normal-appearing (orange). The black vertical and horizontal dotted lines indicate the full range for healthy volunteer CBF and K_1_ values, respectively.

Region of interest data in patients showed a clear relationship between CBF and glucose transport (as measured by K_1_) across all tissue classes (R^2^ = 0.65, *P* < 0.001. Fig. 3). This relationship was driven by core and penumbra (R^2^ = 0.81 and 0.48, respectively, *P* < 0.001), where both K_1_ and CBF were below the lower limit of values found within healthy volunteers. There was no significant relationship within peri-penumbra and normal-appearing tissue (both R^2^ = 0.1, *P* = 0.08 and *P* = 0.07, respectively).

**Figure 4:**
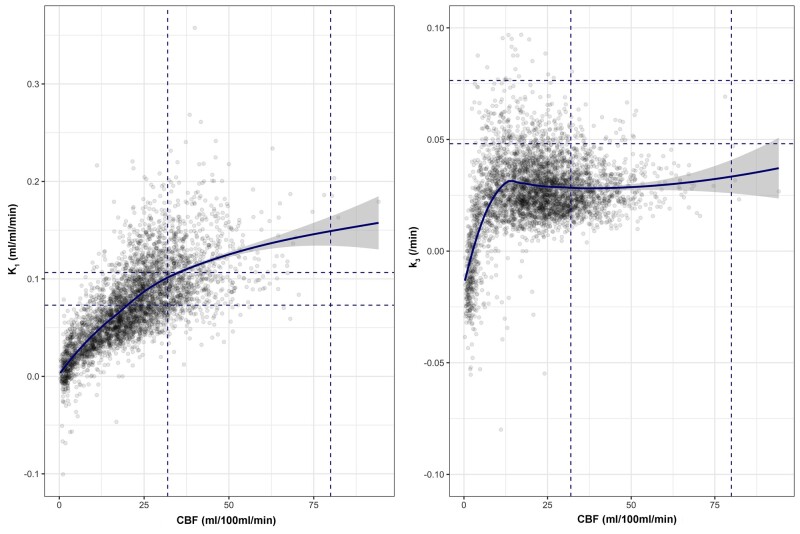
**Relationships between ^18^F-FDG kinetic parameters and CBF based on voxel data.** For each PET imaging session in patients the relationship between CBF and ^18^F-FDG transport (K_1_; *left*) and phosphorylation activity (k_3_; *right*) is plotted for voxels across the whole brain. The fitted light blue lines represent modelling of the relationship between each parameter using locally weighted scatterplot smoothing (LOWESS), with the 95% confidence interval shown in grey. The blue vertical and horizontal dotted lines indicate the full range for healthy volunteer CBF and ^18^F-FDG kinetic parameters, respectively.

Whole-brain voxel-wise analysis showed that where CBF was maintained within the normal range, K_1_ was higher than within healthy volunteers, but reductions in CBF below 25 ml/100 ml/min were associated with steep reductions in K_1_ (Fig. 4 and Supplementary Fig. 7).

### Glucose utilization

#### Regional glucose utilization and phosphorylation rates are discordant and variable

Data are displayed in Table 2. There was a general decrease in phosphorylation activity (k_3_) in TBI patients with normal-appearing brain showing lower activity than healthy volunteers 0.029 (0.024–0.034) versus 0.057 (0.055–0.063)/min, *P* < 0.001. In patients, k_3_ was highest within core 0.044 (0.036–0.057)/min with values significantly higher than peri-penumbra 0.030 (0.025–0.037)/min and normal-appearing brain (*P* < 0.001 for both comparisons). Penumbra k_3_ values 0.039 (0.033–0.043)/min were higher than normal-appearing brain (*P* = 0.002). Increases in k_3_ were associated with a fall in metabolic ratio, particularly within core and penumbra where metabolic ratio was <4 (Table 2 and Supplementary Fig. 8).

Glucose metabolism and K_i_ were lowest in core and highest in normal-appearing brain, but significantly lower when compared to healthy volunteers (*P* < 0.001 for both comparisons). When dephosphorylation (k_4_) was included within regional kinetic modelling the results were similar (Supplementary Table 2). A similar pattern was found for CBF, CBV, CMRO_2_ and metabolic ratio, with lowest values found within the core, and greater values found with increasing distance from the lesion core. The temporal pattern of regional data is displayed in Fig. 2 and Supplementary Table 1. There was a non-significant trend for higher k_3_ values at late time points post-injury within core (*P* = 0.09), and higher metabolic ratio values within all regions of interest at early time points (which did not survive correction for multiple comparisons). In comparison, regional CMRO_2_ and OEF showed higher values at early times points post-injury, with CMRO_2_ values for normal-appearing brain (*P* = 0.003) and OEF values in penumbra (*P* = 0.004) remaining significant after correction for multiple comparisons.

#### Phosphorylation activity is dependent on the adequacy of cerebral blood flow

The voxel-wise relationship between k_3_ and CBF showed variability both within and between subjects (Supplementary Fig. 7), but demonstrated clear patterns across the dataset. k3 remained constant across the range of CBF values found in healthy volunteers, but showed a small rise at CBF ∼20 ml/100 ml/min, followed by a precipitous fall with CBF <12 ml/100 ml/min (Fig. 4). A similar relationship was seen between k_3_ and CMRO_2_ (Supplementary Fig. 9).

#### Relationships between plasma and microdialysis glucose and phosphorylation activity

Across all structural regions in patients, a lower plasma glucose was associated with an increase in k_3_ and K_i_ (Rho = −0.33 and −0.50, respectively, *P* < 0.001). Using the modelled ^18^F-FDG kinetic parameters, we calculated the proportion of brain tissue glucose phosphorylated across all patient regions and related this to plasma and calculated brain glucose concentration. The fraction of tissue glucose undergoing phosphorylation increases when plasma glucose falls to <6–8 mmol/l and/or brain glucose <1–2 mmol/l, particularly within the vicinity of brain lesions (Supplementary Fig. 10).

For 10 PET sessions within eight patients we found microdialysis glucose of 1.6 (0.9–2.8) mmol/l, lactate 4.4 (1.9–6.5) mmol/l, pyruvate 138.4 (95.8–181.9) µmol/l, glutamate 10.4 (4.2–22.4) µmol/l and the lactate/pyruvate ratio 28.3 (17.4–49.3). Lower microdialysis glucose was associated with an increase in k_3_ (Rho = −0.73, *P* = 0.02), (Supplementary Fig. 11) as well as K_i_ (Rho = −0.71, *P* = 0.03). There were no significant relationships between microdialysis glucose and glutamate, pyruvate, lactate or lactate/pyruvate ratio. No microdialysis catheters were inserted in areas where k_3_ or OEF values were above hotspot or IBV thresholds.

### Hyperglycolysis is found in perilesional and normal brain, and is largely independent of ischaemia

#### Cerebral physiology differed between k_3_ hotspots and the ischaemic brain volume

**Figure 5:**
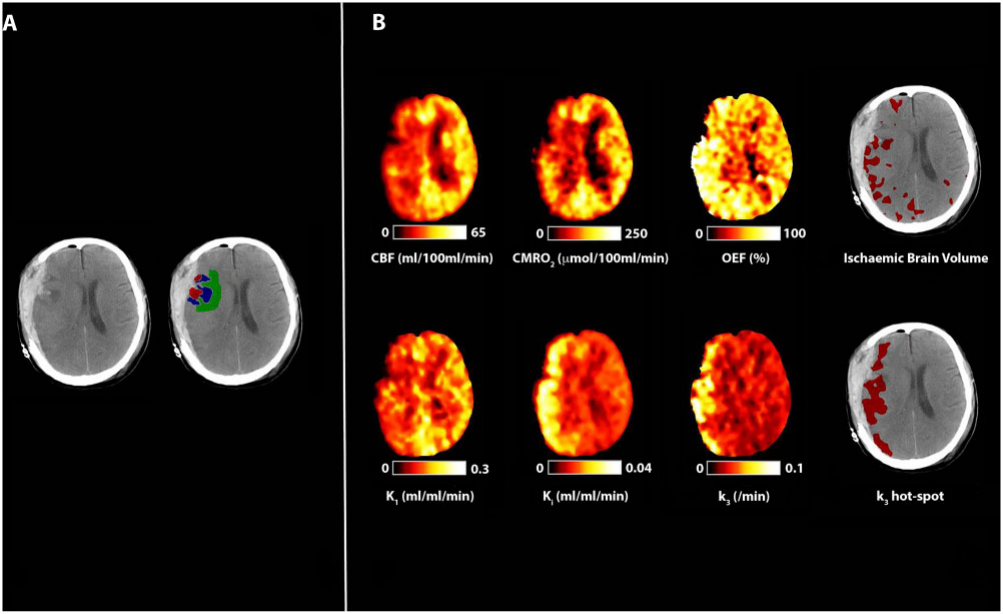
**Evidence of cerebral ischaemia and hyperglycolysis.** CT, CBF, CMRO_2_, OEF, and ^18^F-FDG kinetic parameters obtained in a 40-year-old female within 24 h of severe TBI resulting from a fall. Imaging was obtained following evacuation of a subdural haematoma. Plasma glucose during PET was 3.7 mmol/l. (**A**) CT demonstrates residual subdural blood with minimal midline shift and underlying haemorrhagic contusions. Regions of interest are defined for haemorrhagic lesion (core, red), hypodense tissue (penumbra, blue), and a 1 cm border zone of normal-appearing tissue (peri-penumbra, green). (**B**) Co-registered parametric maps of CBF, CMRO_2_, OEF, K_1_, K_i_ and k_3_. The regions with a critical increase in OEF above the individually calculated threshold (ischaemic brain volume) and increased phosphorylation activity (k_3_ ‘hotspot’) are both shown in red overlying the CT scan. The ischaemic brain volume and k_3_ hotspot volume were 96 ml and 174 ml, respectively, and the volume of overlap between these two tissue classes in this patient was 38 ml.

The volume of brain meeting the criteria for a k_3_ hotspot was larger than IBV: 64 (55–96) versus 7 (2–19) ml (*P* < 0.001, Mann-Whitney U-test), and the volume of k_3_ hotspot and IBV did not differ between early, intermediate and late groups. Similar proportions of k_3_ hotspots [34 (13–44)%] and IBV [29 (15–54)%] were within 1 cm of a lesion (*P* = 0.75), but regions of physiological derangement consistent with both tissue classes were seen within normal-appearing brain. Evidence of overlap between k_3_ hotspots and IBV was uncommon [0 (0–1) ml], with a Dice similarity co-efficient of 0.01 (0–0.02), and even where such overlap was substantial, it represented the minority of each physiological tissue class (Fig. 5). More commonly, k_3_ increases were found in the absence of ischaemia, both within the vicinity of injured (Fig. 1) and normal-appearing brain (Supplementary Fig. 12).

The underlying physiology in k_3_ hotspots and the IBV is summarized in Supplementary Table 3. Despite lower CBF in k_3_ hotspots [25.0 (21.9–29.5) ml/100 ml/min] compared to healthy volunteers [42.9 (37.0–48.5)] and normal-appearing brain in patients [28.6 (26.4–31.4)], there was a signature of maintained K_1_ and higher CMRG. Metabolic ratio in k_3_ hotspots [2.7 (2.1–3.3)] was lower than that found within IBV [5.2 (4.0–5.8)] and normal-appearing brain in patients [4.0 (3.5–4.6)].

The temporal pattern of changes in physiology within k_3_ hotspots and the IBV are shown with Supplementary Fig. 13 and Supplementary Table 4. Within k_3_ hotspots, CMRO_2_, OEF and metabolic ratio tended to reduce with time post-TBI, but only comparisons for OEF survived correction for multiple comparisons (*P* = 0.002).

### Association between the ischaemic brain volume and k_3_ hotspots and outcome

Within 24 h, IBV showed a non-significant trend with worse outcome quantified using the GOS (Rho = −0.51, *P* = 0.11), and there was no association across the whole cohort (Rho = −0.28; *P* = 0.13). The volume of k_3_ hotspot was not associated with the GOS (Rho = −0.18, *P* = 0.31).

**Figure 6:**
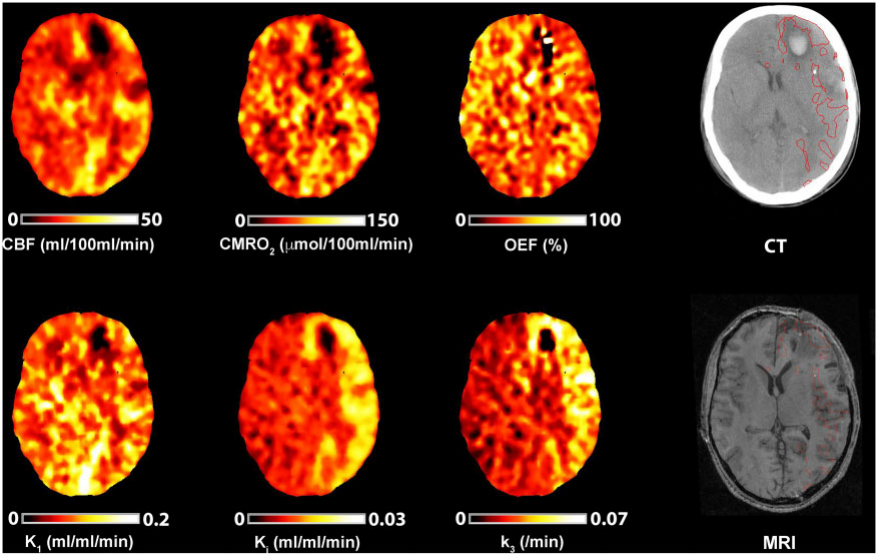
**Association between early metabolic derangements and tissue fate.** Imaging obtained in a 17-year-old male with moderate TBI following a road traffic accident with initial GCS of 12, but who required sedation and ventilation for control of raised ICP. Co-registered CBF, CMRO_2_, OEF, ^18^F-FDG kinetic parameters and CT imaging obtained on Day 8 following TBI. Plasma glucose during imaging was 5.4 mmol/l. The CT demonstrates left frontal and temporal haemorrhagic contusions and is shown with the T_1_-weighted MRI obtained at 9 months post-injury; the MRI has been non-rigidly registered to the CT. Both structural images are displayed with the region of k_3_ hotspot outlined in red, but both lesion core and penumbra have been excluded from the hotspot to ensure that only increases in k_3_ outside of the lesion identified on CT imaging are shown. The volume of brain within the k_3_ hotspot in this subject was 149 ml and, in comparison with brain that appeared structurally normal, had CBF 19.6 versus 19.6 ml/100 ml/min, CMRO_2_ 55.2 versus 60.2 µmol/100 ml/min, OEF 37.8 versus 42.4%, K_1_ 0.097 versus 0.087 ml/ml/min, K_i_ 0.018 versus 0.012 ml/ml/min and k_3_ 0.058 versus 0.024/min, respectively. The T_1_-weighted MRI demonstrates established lesions within the left frontal and temporal brain regions in close proximity with k_3_ increases outside of lesion core and penumbra identified on the CT image.

Eight patients underwent follow-up MRI at >3 months post-injury (median 9, range 4–35 months), of which seven had T_1_-weighted volumetric images for comparison with similar data acquired within the nine ^18^F-FDG PET healthy volunteers. Mean normalized cortical grey matter volume was similar (0.42 versus 0.39, *P* = 0.05) in patients and healthy volunteers, while white matter volume was lower in patients (0.42 versus 0.47, *P* < 0.01). The mean Jacobian determinant values for cortical grey and white matter regions in patients compared with healthy volunteers were 0.04 versus −0.01 (*P* = 0.01) and 0.02 versus −0.01 (*P* = 0.06), respectively, with higher values indicative of smaller volumes requiring expansion to match the brain template. Early increases in k_3_ outside of lesion core and penumbra were often found in close proximity to areas showing evidence of late structural injury on MRI obtained at follow-up (Fig. 6). There was no consistent voxel-wise relationship between k_3_ and volume contraction using Jacobian determinant values across the normal-appearing brain in TBI patients (Supplementary Fig. 14).

## Discussion

We used ^18^F-FDG and multi-tracer ^15^O PET together with brain microdialysis in a cohort of acute TBI patients to image the impact of glucose availability, delivery and utilization on cerebral metabolism. Brain glucose delivery (K_1_) is dependent on both plasma glucose and the adequacy of CBF, particularly within regions where CBF <25 ml/100 ml/min. An overall global reduction in glucose utilization following TBI appears to be driven by low glucose delivery and low phosphorylation activity, but regional hotspots were found within, and distant from, visible brain lesions. Such increases were explained by preserved glucose delivery (K_1_) despite reductions in CBF, and in plasma and brain glucose. Increases in glucose utilization were typically accompanied by CMRO_2_ reductions, but without OEF increases consistent with ischaemia. These metabolic derangements represent a potential therapeutic target.

### Glucose availability, delivery and transport

We confirm previous findings that brain glucose is associated with serum glucose, but variable across the injured brain,^8^^,^^31^ and that K_1_ is similar to healthy volunteers in normal-appearing brain and reduced around lesions following TBI.^2^ However, we also show for the first time, that K_1_ was related to CBF, particularly at CBF <25 ml/100 ml/min (Figs  3 and 4). Microdialysis shows a linear relationship between plasma and brain glucose in non-injured areas after TBI,^8^^,^^31^ but glucose transport is impaired within the injured brain.^8^ CBF may be the rate-limiting factor for glucose transport into the brain, but we found that normal-appearing brain in TBI patients had similar K_1_ to that found within healthy volunteers despite lower CBF, suggesting that increases in GLUT-1 expression may also contribute.^32^

### Glucose utilization

In comparison with healthy volunteers, TBI patients showed a global decrease in glucose utilization, but derangements in kinetic parameters were heterogeneous across the injured brain, in keeping with past reports,^33^ with reductions attributed to a combination of sedation,^34^^,^^35^ decreased consciousness and/or TBI itself.^36^ All patients who underwent PET imaging received variable rate infusions of sedatives and analgesics to facilitate metabolic suppression as part of intensive care management and ICP-directed therapy, but despite this, there were regional increases in glucose utilization. The metabolic ratio was reduced within visible brain lesions, but also within normal-appearing brain, as reported previously.^15^^,^^37^ Lower values within lesion core and penumbra imply increased anaerobic glycolysis, but the lack of OEF increase is against classical ischaemia and is suggestive of microvascular ischaemia and mitochondrial dysfunction.^7^

Whilst visible lesions had the lowest K_i_ and CMRG, this was driven by low glucose delivery (K_1_ and CBF) rather than a reduction in k_3_, which was higher than that found within normal-appearing brain. A sharp fall in k_3_ was associated with CBF <12 ml/100 ml/min and cerebral oxygen metabolism consistent with irreversible tissue injury (<40 µmol/100 ml/min).^38^ Prior to reaching this threshold, increases in k_3_ within individual subjects (Supplementary Fig. 7) were seen below the lower limit of CBF found in healthy volunteers (<25 ml/100 ml/min). Such findings support measures to preserve or optimize CBF and glucose delivery within the vicinity of brain lesions to maintain metabolism and prevent neuronal loss. In addition, studies highlight that alternative substrates may be utilized as an energy source, via the tricarboxylic acid cycle, following TBI and are a potential therapeutic approach.^39–42^

Increases in k_3_ and K_i_ in TBI patients were associated with both lower plasma glucose and microdialysis glucose, raising the possibility that low brain glucose is driven by increased consumption as well as reduced delivery. Our data show that the proportion of brain glucose utilized increases as plasma and brain glucose falls below 6–8 and 1–2 mmol/l, respectively, particularly within the vicinity of lesions (Supplementary Fig. 10). Other studies have shown CMRG increases with tight glycaemic control, associated with an increase in lactate/pyruvate ratio indicative of metabolic stress.^43^ We found no association between microdialysis lactate or lactate/pyruvate ratio and k_3_ in our current study, and have previously shown an increase in glucose metabolism to both lactate and pyruvate with no change in the lactate/pyruvate ratio following TBI.^44^ Randomized controlled trials of intensive insulin therapy in TBI show no benefit,^45–47^ and though a recent meta-analysis is inconclusive,^48^ our data suggest that plasma glucose levels should be carefully controlled and not be allowed to fall below 6–8 mmol/l, ideally in combination with monitoring of brain glucose to ensure values of 1–2 mmol/l are maintained across the injured brain.

Previous ^18^F-FDG PET studies show hyperglycolysis following TBI,^12^ driven by regional phosphorylation increases.^2^ We delineated the burden and spatial pattern of k_3_ hotspots and compared this with evidence of ischaemia (IBV). The k_3_ hotspot regions were found both in the vicinity of, and distant from visible lesions. The volume of k_3_ hotspots was larger than the IBV, the underlying physiology different, and there was little spatial overlap between these two tissues classes. While ischaemia and hyperglycolysis sometimes overlapped within 24 h of injury (Fig. 5), this was uncommon. The typical metabolic signature of a k_3_ hotspot was higher CMRG with preserved K_1_ and OEF, despite lower CBF, CMRO_2_ and metabolic ratio. A late (Day 6–12 post-TBI) trend for lower CMRO_2_, OEF and metabolic ratio within k_3_ hotspots was suggestive of worsening or persistent metabolic derangements and risk of neuronal loss with CMRO_2_ values close to the threshold for tissue viability (<40 µmol/100 ml/min).^38^ While such changes could reflect differences in intensive care management at different phases of recovery following TBI, all received standardized care. This pattern of metabolic derangements is also consistent with microvascular ischaemia^7^ and/or mitochondrial dysfunction,^49^ and could be associated with such pathophysiological processes as spreading depolarization,^50^ cytotoxic oedema,^51^ and neuroinflammation.^52^ Such processes are common following TBI,^7^ and are consistent with our finding of a low metabolic ratio secondary to a decrease in oxygen metabolism within regions showing increases in glucose utilization. In patients, follow-up imaging of brain metabolism, with structural and functional tissue outcome evaluated using MRI, and functional outcome assessments would be required to definitively address the significance of early metabolic derangements.

We compared ^18^F-FDG PET obtained within a group of TBI patients to identical data obtained in healthy volunteers using standard kinetic modelling.^53–55^ Previously published values for ^18^F-FDG kinetic parameters in healthy volunteers and TBI patients^2^^,^^56^ are similar to the data we report, and the available test-retest reproducibility data suggest a co-efficient of variation of up to 10–15% for glucose metabolism.^57^ While test-retest variability is important in assessing the significance of interventional or serial studies within the same subject, in this observational study we demonstrate regional changes in ^18^F-FDG kinetic parameters and glucose metabolism between TBI subjects and healthy volunteers of up to 50% or more across the injured brain. At the voxel level, to avoid high variability in the ^18^F-FDG kinetic parameters, we used a basis function version of a two-tissue compartment model, which assumes irreversible phosphorylation of ^18^F-FDG.^22^^,^^54^^,^^58^ Modelling the data on a regional basis with the inclusion of dephosphorylation (k_4_) showed similar results (Supplementary Table 2). In contrast to Hattori *et al*.,^2^ we found no differences in k_4_ between patient regions on interest, or in comparison with healthy volunteers. The lumped constant within normal-appearing brain in TBI patients (0.41) was lower than that found within healthy volunteers (0.56), and is comparable to previous reports.^36^ While this value is used to calculate CMRG, we also report the influx rate constant (K_i_).

### Outcome impact

We have previously shown that early ischaemia (<24 h post-TBI) was associated with poor outcome.^17^ We were unable to confirm this finding, perhaps because IBV was not increased within 24 h of injury in the current cohort. k_3_ hotspots were found throughout the first 12 days post-TBI, but there was no association with poor outcome, as defined by GOS. Those patients who underwent follow-up MRI demonstrated lower normalized global white matter volume compared with healthy volunteers and a trend for lower cortical grey and white matter volumes. While k_3_ hotspots in structurally normal brain (on CT) may be associated with MRI visible lesions at follow-up, the relationship between k_3_ and volume contraction was variable. Metabolic derangements resulting in both increases and decreases in k_3_ may be associated with late structural injury and further study is required to confirm the significance of these derangements.

### Limitations

We examined the impact of TBI on glucose delivery and metabolism using PET obtained within a typical patient cohort, rather than examine the temporal pattern and evolution within individual subjects. As such, we did not acquire follow-up MRI in all subjects and the analyses of the possible relationship between early metabolic derangements and structural outcome presented are illustrative of how future studies could address this issue. Future sequential imaging studies incorporating MRI and spectroscopy,^59^ diffusion tensor imaging and multi-tracer PET (^15^O, ^18^F-FDG, and SV2A)^60^ could delineate evidence of pan-necrosis, cortical atrophy and selective neuronal loss related to early metabolic derangements, and correlate these with functional deficits resulting in poor outcome post TBI.

Acute MRI was not available for TBI patients and evidence of structural injury was defined on the basis of early CT. While we may have missed structural changes not visible on CT, we were able to demonstrate significant physiological differences between structural regions of interest and used PET to define metabolic regions of interest for analyses.

## Conclusions

The data presented in this study confirm previous work^61^ and show that, up to 12 days post-injury, there is an overall reduction in oxygen and glucose metabolism across the brain. Our results show that K_1_ is preserved within normal-appearing brain in the face of CBF reduction and this may help maintain glucose delivery, and mitigate the impact of modest plasma glucose reductions. However, this compensation fails when CBF and/or glucose delivery are critically low. Despite this, such regions can show high k_3_ values, providing evidence of metabolically active tissue, particularly within the vicinity of brain lesions at risk of expansion. Regions of increased glucose utilization were associated with non-ischaemic reductions in CBF and CMRO_2_ consistent with microvascular ischaemia and/or mitochondrial dysfunction. The primary physiological target in TBI has been the maintenance of CBF to ensure oxygen delivery, but we show that it may be just as important to ensure adequate glucose delivery. Where CBF elevation is either impossible or ineffective, our data provide a physiological framework for maintaining glucose at high normal levels (8–10 mmol/l) to optimize substrate delivery. Finally, these data make a case for the development and evaluation of alternative substrates (such as ketones) whose delivery is independent of transporters used to deliver glucose.^42^

## Supplementary Material

awab255_Supplementary_Data

## Abbreviations

CBF: cerebral blood flow
CBV: cerebral blood volume
CMRG: cerebral glucose metabolism
CMRO_2_: oxygen metabolism
GOS: Glasgow Outcome Score
IBV: ischaemic brain volume
ICP: intracranial pressure
OEF: oxygen extraction fraction
TBI: traumatic brain injury
