# Supplementary material for: Metabolic derangements are associated with impaired glucose delivery following traumatic brain injury
Source: Brain. 2021 Jul 8;144(11):3492–504. doi: 10.1093/brain/awab255 (PMC8677561; doi:10.1093/brain/awab255)
Supplement: awab255_Supplementary_Data [file awab255_supplementary_data.pdf]

Metabolic derangements are associated with impaired glucose delivery following traumatic brain injury

Jeroen Hermanides, Young T Hong, Monica Trivedi, Joanne Outtrim, Franklin I Aigbirhio, Peter J Nestor, Matthew Guilfoyle, Stefan Winzeck, Virginia FJ Newcombe, Tilak Das, Marta M Correia, Keri LH Carpenter, Peter JA Hutchinson, Arun K Gupta, Tim D Fryer, John D Pickard, David K Menon, Jonathan P Coles

Corresponding author: Dr JP Coles, Division of Anaesthesia, Department of Medicine, University of Cambridge, Addenbrooke's Hospital, Cambridge, UK. Tel 00441223217889, e-mail jpc44@cam.ac.uk

Supplementary material

Materials and methods

Imaging

Positron emission tomography data were acquired in 3D mode for the last 10 minutes of a 20-minute steady state infusion of 800 MBq of ^{15}O water; in 3D mode for 5 minutes after a 1 minute inhalational of 750MBq of ^{15}O carbon monoxide; and in 2D mode for the last 10 minutes of a 20-minute steady state inhalation of 7,200 MBq of ^{15}O oxygen. Following intravenous administration of 74 MBq ^{18}F-FDG (injected over 30 seconds) dynamic scanning in 3D mode was acquired for a further 55 minutes. Emission data were corrected for photon attenuation using data from a 10-minute transmission scan with rotating germanium-68 rods and a corresponding 60-minute blank scan. Images were reconstructed into 2.34×2.34×4.25 mm voxels using the PROMIS 3D filtered back projection algorithm,[1] with corrections applied for randoms, dead time, normalisation, scatter, attenuation, and sensitivity. Data were smoothed using an isotropic 4 mm Gaussian filter. For ^{15}O PET, parametric maps of cerebral

blood flow (CBF), blood volume (CBV), oxygen metabolism ($CMRO_2$), and oxygen extraction fraction (OEF) were calculated by inputting image and arterial activity concentration measurements into standard models.[2,3] We used a blood-brain partition coefficient for ^{15}O water of 0.95 based upon the previous in vitro data[4] and a small to large vessel haematocrit ratio of 0.85.[5] For ^{18}F-FDG, voxel-wise kinetic modelling used a basis function version[6] of the irreversible two-tissue compartment model originally published by Sokoloff et al[7] (Supplementary Fig. 1) with an arterial plasma input function to calculate K_1, k_2, and k_3. The fitting procedure employed in this study is that described in Hong and Fryer[6], including the number of basis functions and the basis function ranges. From these parameters we calculated the ^{18}F-FDG influx rate constant (K_i) from $K_i = K_1 \times (k_3/k_2+k_3)$. Using the lumped constant (LC)[8] from $LC = 0.3+0.8 \times (K_i/K_1)$ we calculated glucose metabolic rate (CMRG) from $CMRG = PG \times (K_i/LC)$, where PG is plasma glucose (μmol/L). In a separate analysis we modelled the region of interest (ROI) ^{18}F-FDG data using the above irreversible model methodology and a reversible version of the two-tissue compartment model basis function method that included dephosphorylation (k_4);[6] k_4 was not included in the voxel-wise kinetic modelling as it induced high parameter variability. Using factors to convert ^{18}F-FDG kinetic parameters to those of glucose, we calculated brain tissue glucose concentration (C_T) from C_T = PG x (K_1/tau)/[(k_2/tau)+(k_3/phi)], where tau = 1.1 and phi = 0.3.[8,9] In addition, the proportion of brain tissue glucose that is phosphorylated was calculated from (k_3/phi)/[(k_2/tau)+(k_3/phi)].

Image analysis

We estimated an individualised voxel-based critical OEF threshold (OEF_{crit}),[10,11] which equated to a cerebral venous oxygen content (CvO_2) of 3.5 mL/100mL,[12,13] for each subject as follows:

$$OEF_{crit} = \frac{(CaO_2 - 3.5)}{CaO_2}$$

where,

$CaO_2 = 1.34\text{Hb}.\text{SaO}_2 + 0.0225\text{PaO}_2$

CaO_2 is arterial oxygen content, Hb is the haemoglobin concentration in g/100mL, SaO_2 is the fractional arterial oxygen saturation and PaO_2 is the arterial partial pressure of oxygen (kPa).

The accuracy of registration between structural MRI obtained at follow-up and acute CT imaging using ANTs[14] was assessed independently by two experts (VFJN and TD) using 8 anatomical landmarks (calcified pineal, posterior recesses of 4th ventricle, anterior septum pellucidum, left and right tip of frontal horn of lateral ventricle, left and right tip of posterior horn of lateral ventricle, and the midline of the top axial slice of the brain. For each landmark, the maximum difference between CT and MRI in any plane (x, y, or z) was recorded for each subject.

The T1-weighted MRI were processed within a TBI pipeline incorporating neck cropping and correction for scanner field inhomogeneities, with brain parcellation into grey and white matter ROIs performed using multi-atlas label propagation with expectation-maximization–based refinement, which provides robust segmentation even when anatomy is distorted owing to trauma.[15] The volume of cortical grey and white matter ROIs were normalised by total brain volume and compared between TBI patients and healthy volunteers. In addition, these ROIs were applied to the Jacobian determinant images providing the amount of contraction or expansion required to normalise the subjects brain into template space. Data used were obtained from the CamCAN repository (available at http://www.mrc-cbu.cam.ac.uk/datasets/camcan/).[16, 17] In the context of TBI, expansion to warp each voxel to the reference template image was indicative of volume contraction/loss at late follow-up. The

voxel-wise relationship between k_3 and volume contraction Jacobian determinant images within normal appearing brain were explored.

Results

Accuracy of kinetic model fits to regional time-activity curves

As a metric of the quality of the fit of the model to the data, the standard error of the regression (SE) was determined for all regional fits. The SE was determined using,

$$SE = \sqrt{\frac{\sum_{i=1}^{N}\left(Y_i - Y_i^{model}\right)^2}{N-2}}$$

where Y_i is the data, Y_i^{model} is the fitted value, and i is the frame number, which has a maximum of N.

The SE for each time-activity curve (TAC) in kBq/ml was normalised by the mean concentration (kBq/ml) of the TAC to produce percentage standard error, i.e., independent of the magnitude of the tissue radioactivity concentration.

Percentage SE values are given in Supplementary Fig. 2 for the two models used in the study. The quality of the fits was good throughout, with median SE values for each group varying from 2.2% (healthy volunteers; HV) to 3.6% (core) for the irreversible model, and 0.9% (healthy volunteers) to 3.1% (core) for the reversible model. The higher SE values in the core are to be expected due to the poorer statistical quality of the data due to low tracer delivery and/or small volume. To a lesser extent low tracer delivery also affects the other regions in TBI subjects.

Exemplar kinetic model fits to regional TACs are given in Supplementary Fig. 3. For all regions (patients: core, penumbra, peri-penumbra, and normal-appearing; and healthy volunteer) fits

with an SE that approximates the median SE for the region are given for both the irreversible and reversible models. In addition, for the core the fits with the highest SE are shown.

Registration accuracy between follow-up MRI and acute CT imaging

Across both experts the median (range) difference across all landmarks was 1.5 (0.6 – 2.70) mm (see Supplementary Fig. 4).

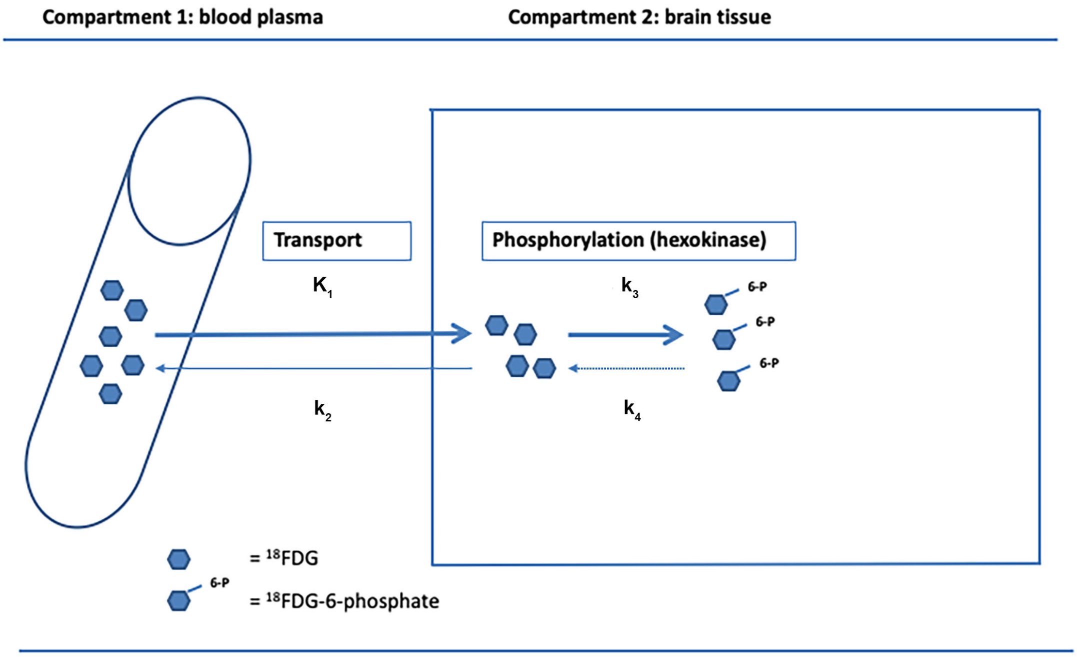

Supplementary Figure 1. Two tissue compartment model for ^{18}F-FDG positron emission tomography

For the purposes of the voxel-wise kinetic modelling we used an irreversible two-tissue compartment model to calculate transport of ^{18}F-FDG into brain tissue (K_1), return to plasma (k_2), and its phosphorylation by hexokinase (k_3). In a separate analysis the region of interest (ROI) ^{18}F-FDG data were modelled using both the irreversible model and a reversible two-tissue compartment model which included dephosphorylation (k_4).

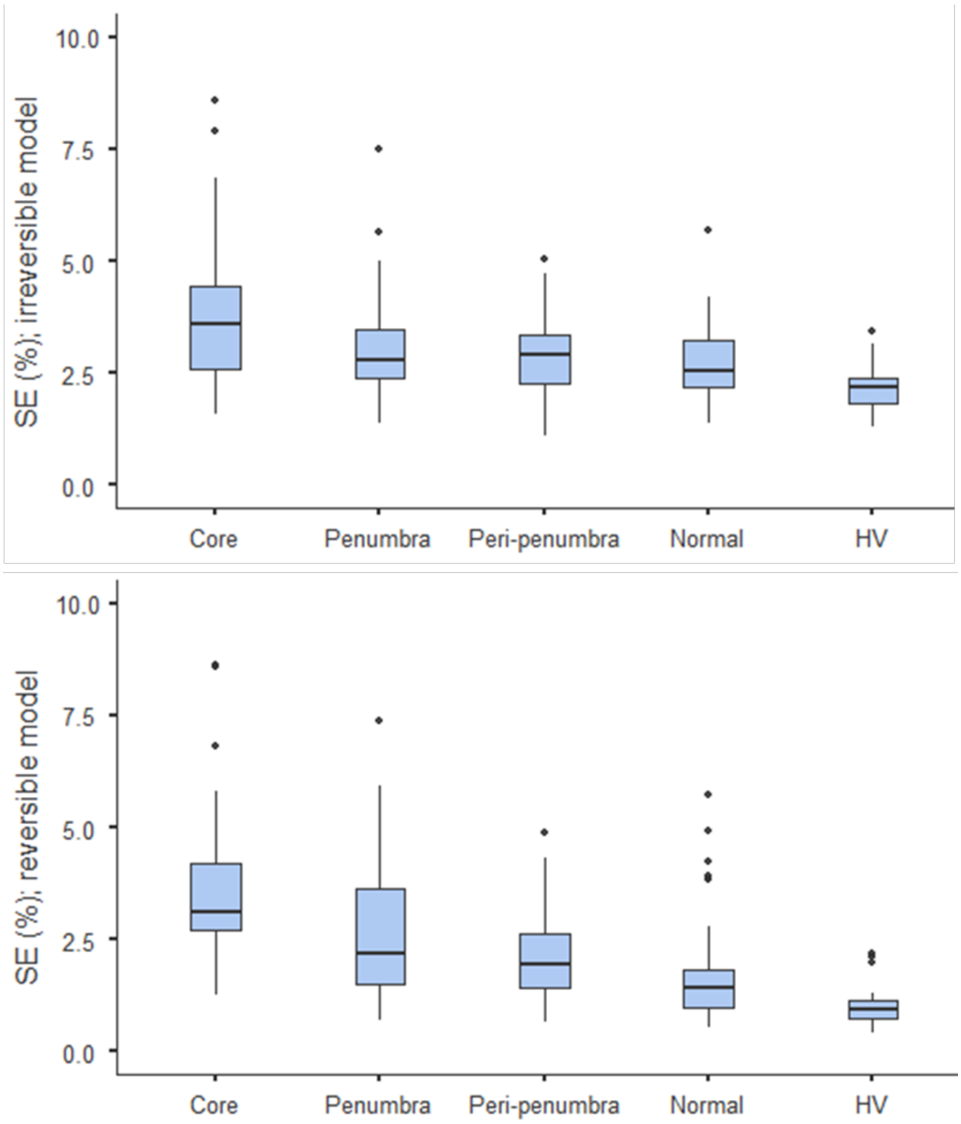

Supplementary Figure 2. Standard error of the regression for regional kinetic model fits

Box plots of percentage standard error of the regression (SE) for kinetic model fits to the patient regions (core, penumbra, peri-penumbra, normal-appearing (Normal)) and the healthy volunteers (HV) for the irreversible model (top) and the reversible model (bottom).

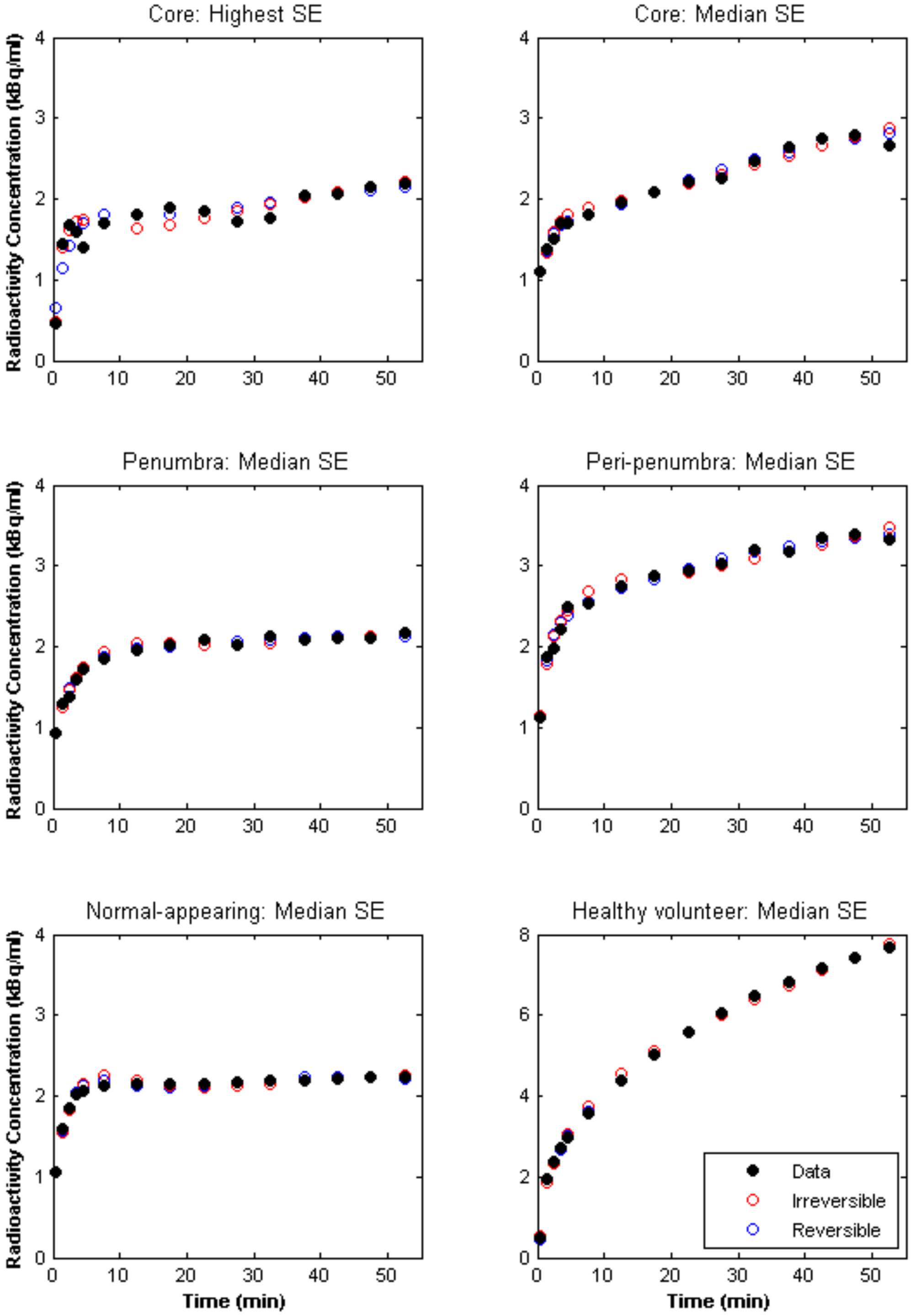

Supplementary Figure 3. Kinetic model fits to regional time-activity curves

Exemplar fits to time-activity curves for patient regions (core, penumbra, peri-penumbra, normal-appearing) and healthy volunteers for the irreversible and reversible models. For each region, fits with a percentage standard error of the regression (SE) that approximates the median SE for that region are given. In addition, for the core the fits with the highest SE are given.

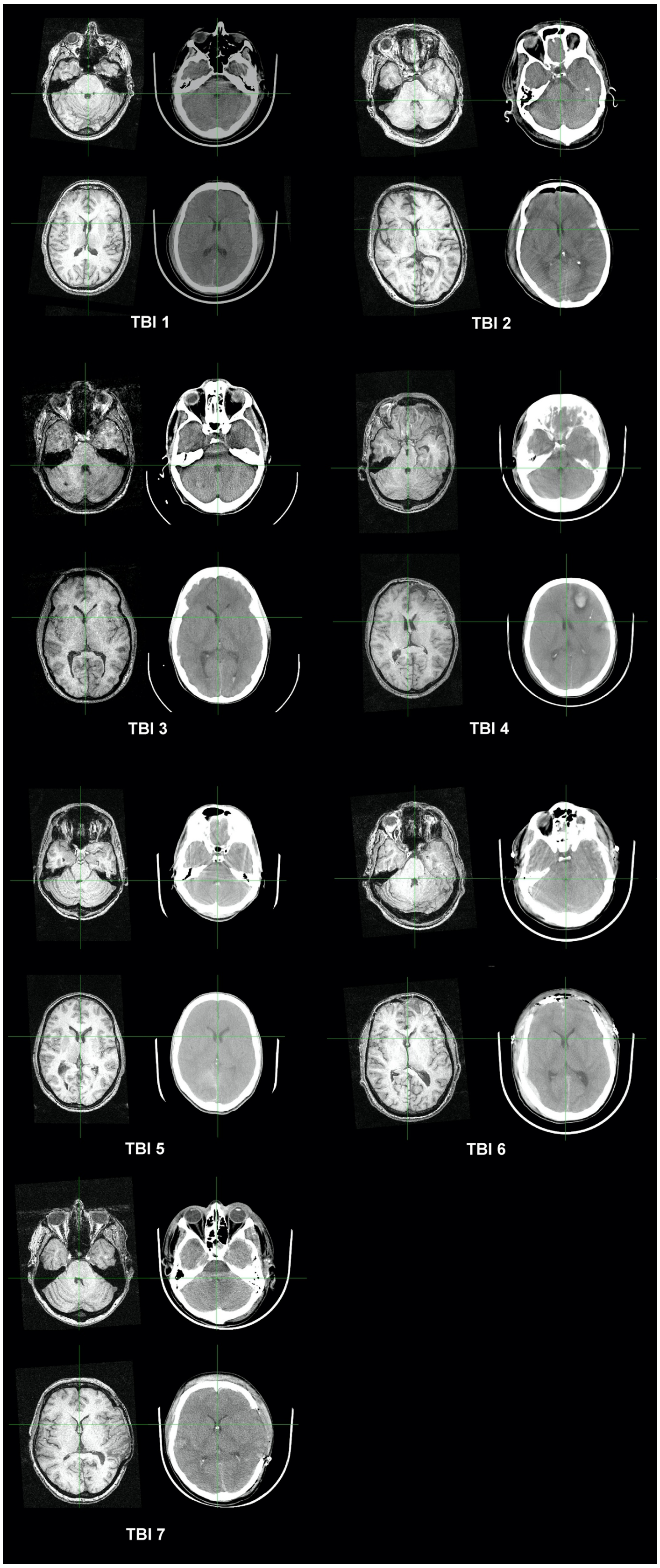

Supplementary Figure 4. Registration of follow-up MRI and acute CT imaging

Seven traumatic brain injury (TBI) patients underwent T1-weighted MRI which were registered to acute CT using Advanced Normalization Tools (ANTs). Two corresponding axial slices are displayed for each subject.

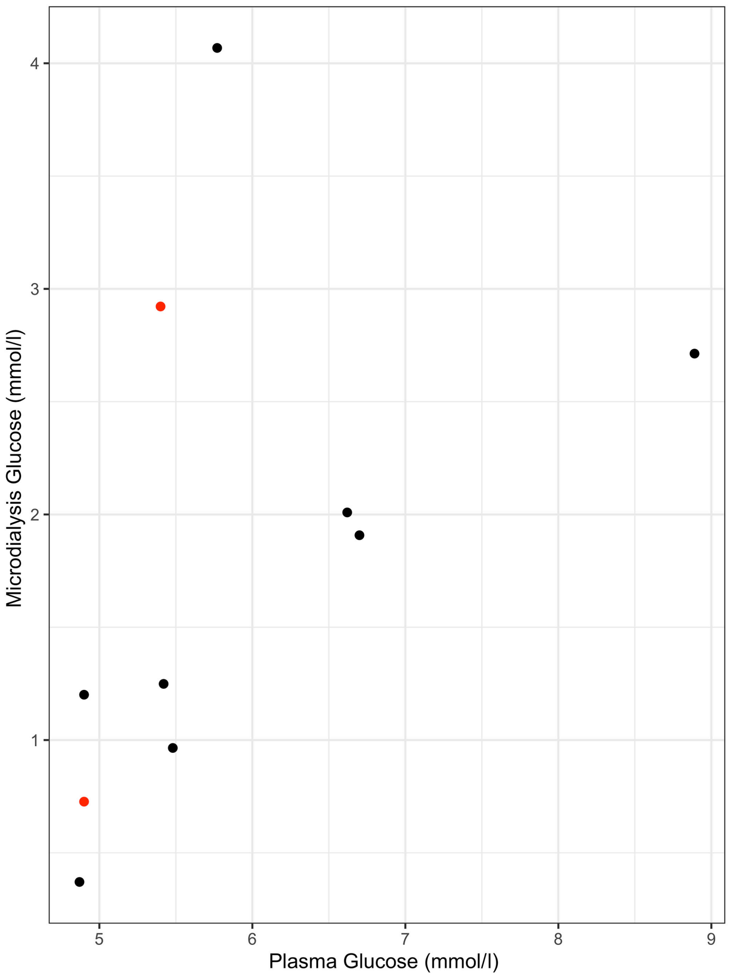

Supplementary Figure 5. Relationship between plasma and microdialysis glucose

Scatterplot of relationship between microdialysis and plasma glucose during 10 PET sessions in 8 patients. The data points highlighted in red indicate the patients where microdialysis catheters were located in close proximity to injured areas.

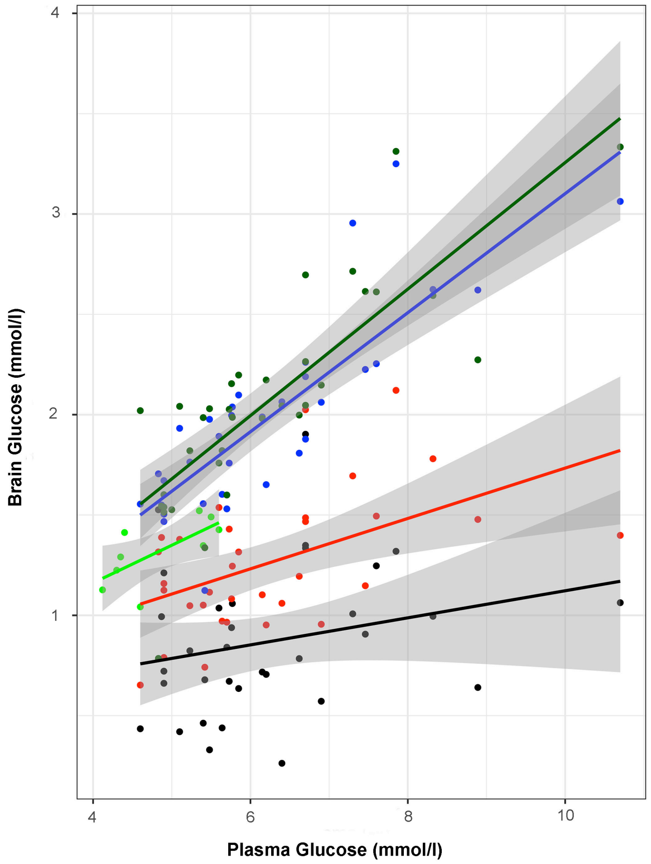

Supplementary Figure 6. Relationship between plasma and calculated brain glucose

Scatterplot of the relationship between plasma and calculated brain tissue glucose concentration in healthy volunteer (light green) and patient regions of interest: lesion core (black), penumbra (red), peri-penumbra (blue) and normal appearing (dark green). The corresponding solid lines are fitted using linear regression for healthy volunteers (R^2 0.47, p=0.04), lesion core (R^2 0.06, p=0.17), penumbra (R^2 0.25, p<0.01), peri-penumbra (R^2 0.69, p<0.001) and normal appearing (R^2 0.66, p<0.001).

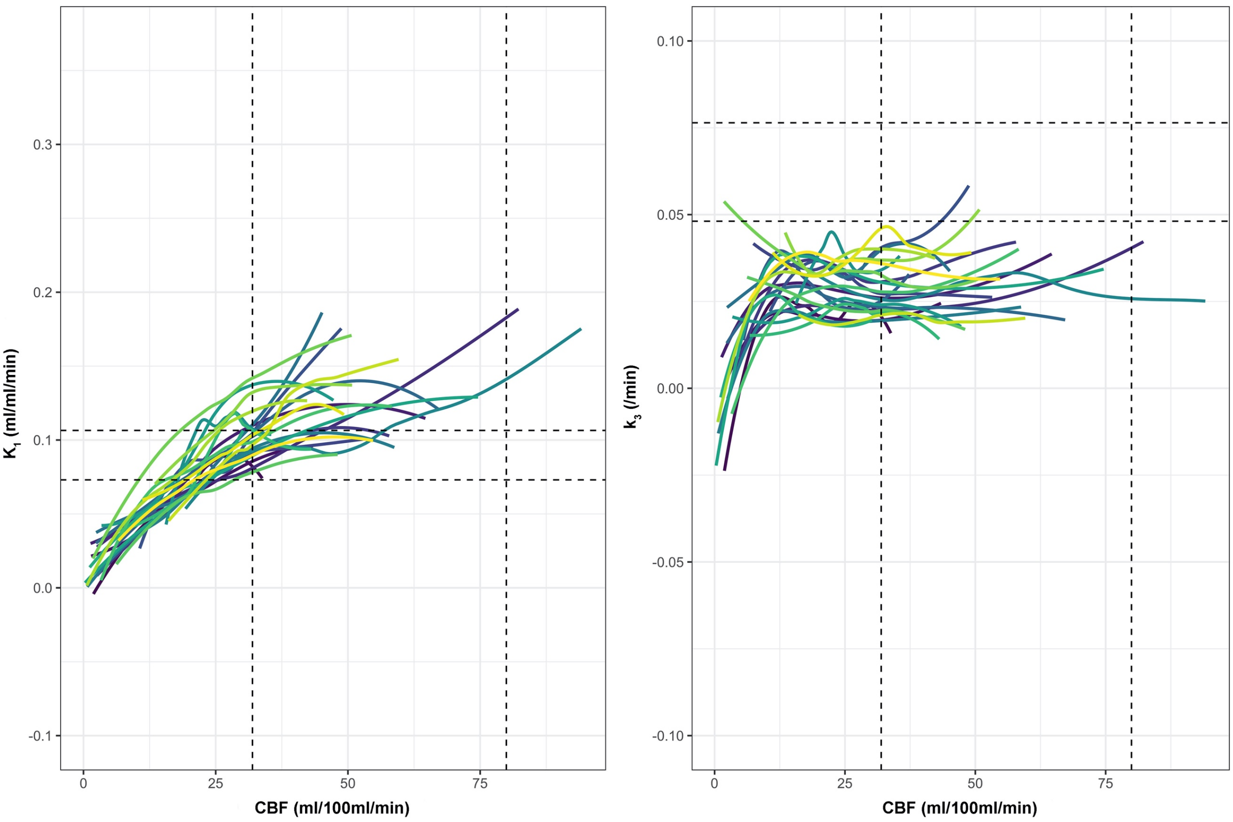

Supplementary Figure 7. Scatterplots of the relationships between cerebral blood flow and ^{18}F-FDG kinetic parameters based on voxel data from individual patients

For each PET imaging session in individual patients the relationship between cerebral blood flow (CBF) and ^{18}F-FDG transport (K_1; left panel) and phosphorylation activity (k_3; right panel) is plotted for voxels across the whole brain using locally weighted scatterplot smoothing. The black vertical and horizontal dotted lines indicate the full range for healthy volunteer CBF and ^{18}F-FDG kinetic parameters respectively.

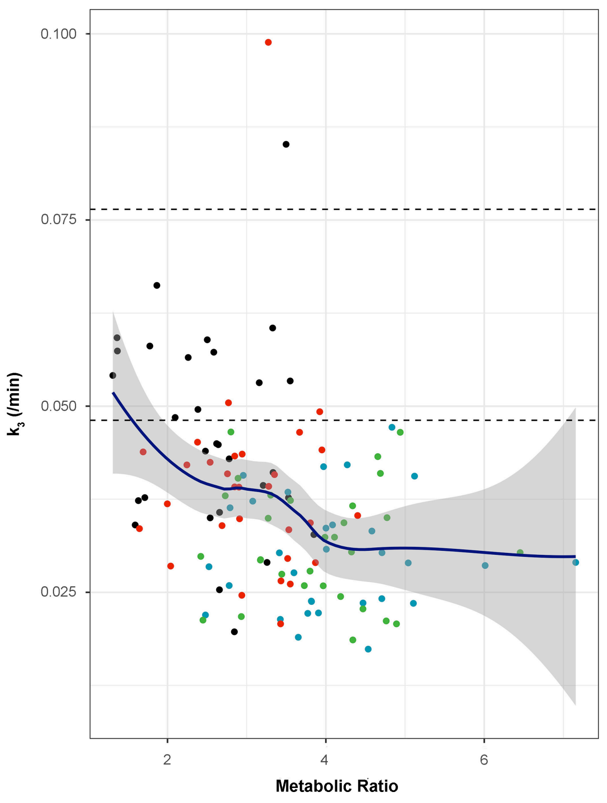

Supplementary Figure 8. Relationship between ^{18}F-FDG phosphorylation activity and metabolic ratio

Scatterplot of relationship between phosphorylation activity (k_3) and metabolic ratio in patient regions of interest: lesion core (black), penumbra (red), peri-penumbra (blue) and normal appearing (green). The fitted blue line represents modelling of the relationship using locally weighted scatterplot smoothing (LOWESS), with the 95% confidence interval shown in grey.

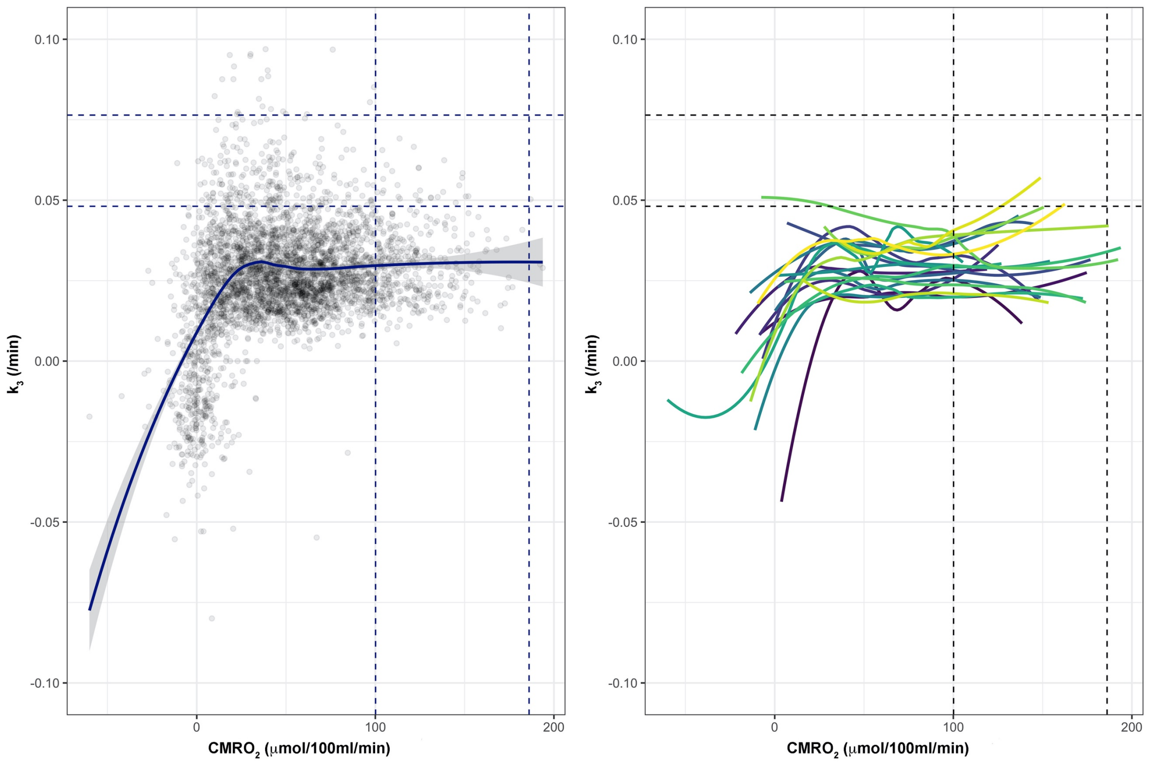

Supplementary Figure 9. Scatterplots of the relationship between cerebral oxygen metabolism and ^{18}F-FDG phosphorylation activity based on voxel data

For each PET imaging session in patients the relationship between ^{18}F-FDG phosphorylation activity (k_3) and cerebral oxygen metabolism ($CMRO_2$) is plotted for voxels across the whole brain using locally weighted scatterplot smoothing for all patients (left panel) and within individual patients (right panel). The blue vertical and horizontal dotted lines indicate the full range for healthy volunteer $CMRO_2$ and k_3 respectively.

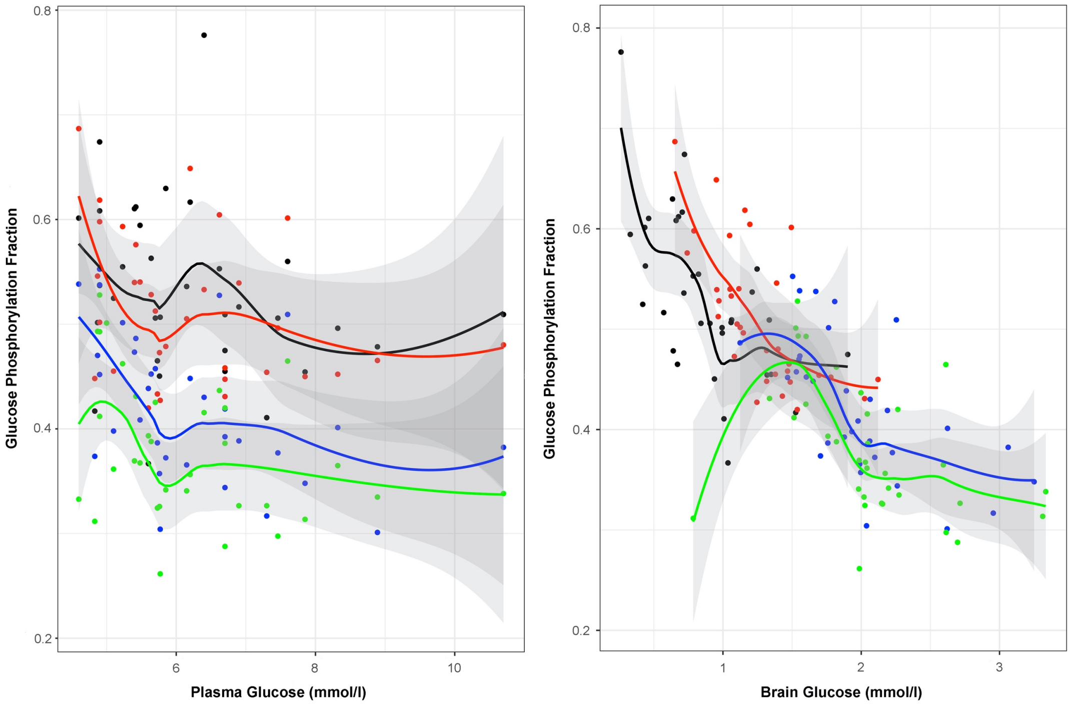

Supplementary Figure 10. The impact of glucose availability on utilisation

Scatterplot of the relationship between the proportion of brain tissue glucose that undergoes phosphorylation and plasma glucose (left panel), and brain tissue glucose (right panel) in patient regions of interest: lesion core (black), penumbra (red), peri-penumbra (blue) and normal appearing (green). The fitted lines represent modelling of the relationship using locally weighted scatterplot smoothing (LOWESS), with the 95% confidence interval shown in grey.

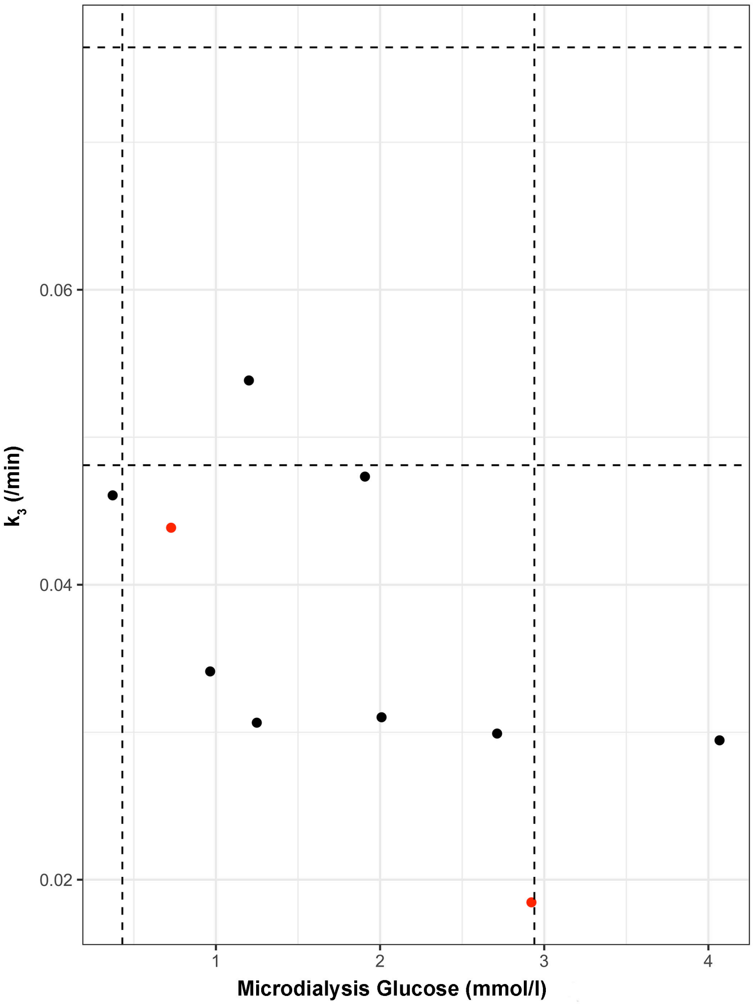

Supplementary Figure 11. Relationship between microdialysis glucose and ^{18}F-FDG phosphorylation

Scatterplot of ^{18}F-FDG phosphorylation (k_3) and microdialysis glucose from 10 PET sessions in 8 patients. The data points highlighted in red indicate the patients where microdialysis catheters were located in close proximity to injured areas. The vertical black dashed lines represent the reference interval (95% confidence interval) obtained from normal-appearing white matter in awake patients following surgery for posterior fossa or supratentorial lesions.[18] The horizontal black dashed lines are the full range of k_3 values found in healthy volunteers.

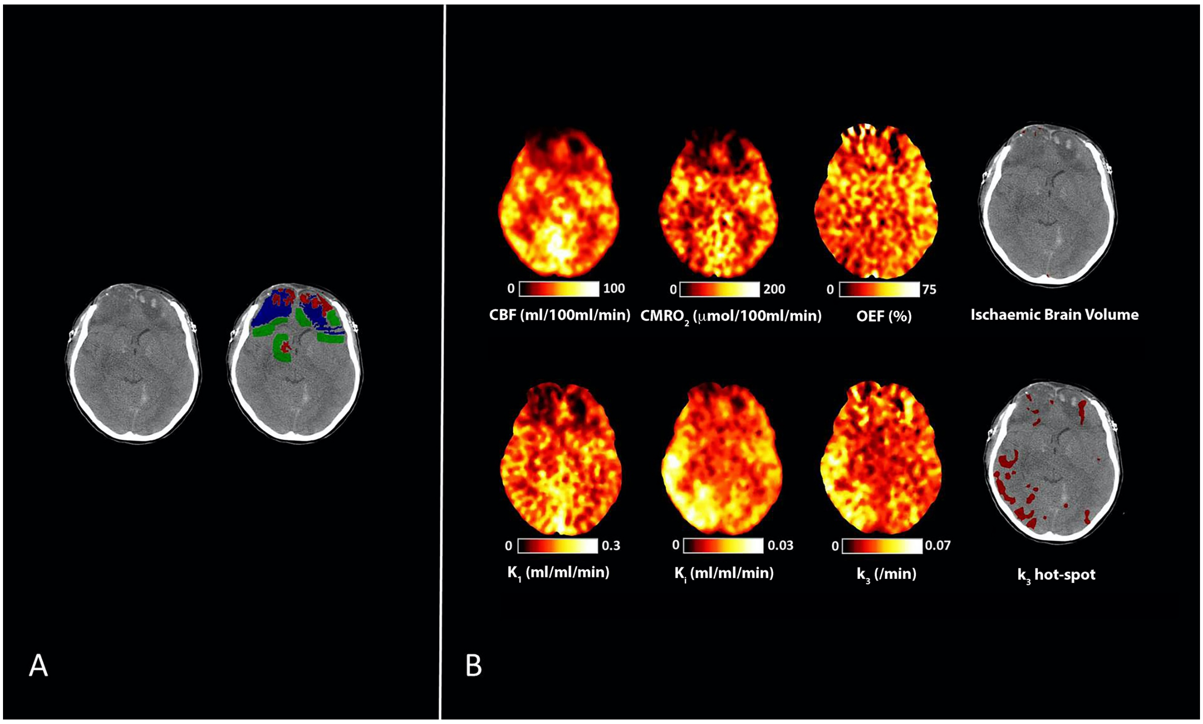

Supplementary Figure 12. Evidence of hyperglycolysis distant from visible lesions

Computed tomography (CT), cerebral blood flow (CBF), oxygen metabolism ($CMRO_2$), oxygen extraction fraction (OEF), and ^{18}F-FDG kinetic parameters obtained in a 36-year old male with severe traumatic brain injury on day four following a road traffic accident. Plasma glucose was 5.8 mmol/L. **A)** CT demonstrates bifrontal hemorrhagic contusions. Regions of interest (ROIs) are defined for hemorrhagic lesion (core, red), hypodense tissue (penumbra, blue), and a 1 cm border zone of normal-appearing tissue (peri-penumbra, green). **B)** Co-registered parametric maps of CBF, $CMRO_2$, OEF, K_1, K_i and k_3. Areas of increased phosphorylation activity (k_3 'hot spots') are shown in red overlying the CT scan in proximity to visible brain lesions, but also in areas that appear structurally normal. The ischemic brain volume and k_3 hot-spot volume were 1 and 78 ml, and there was no overlap between these two tissue classes in this patient.

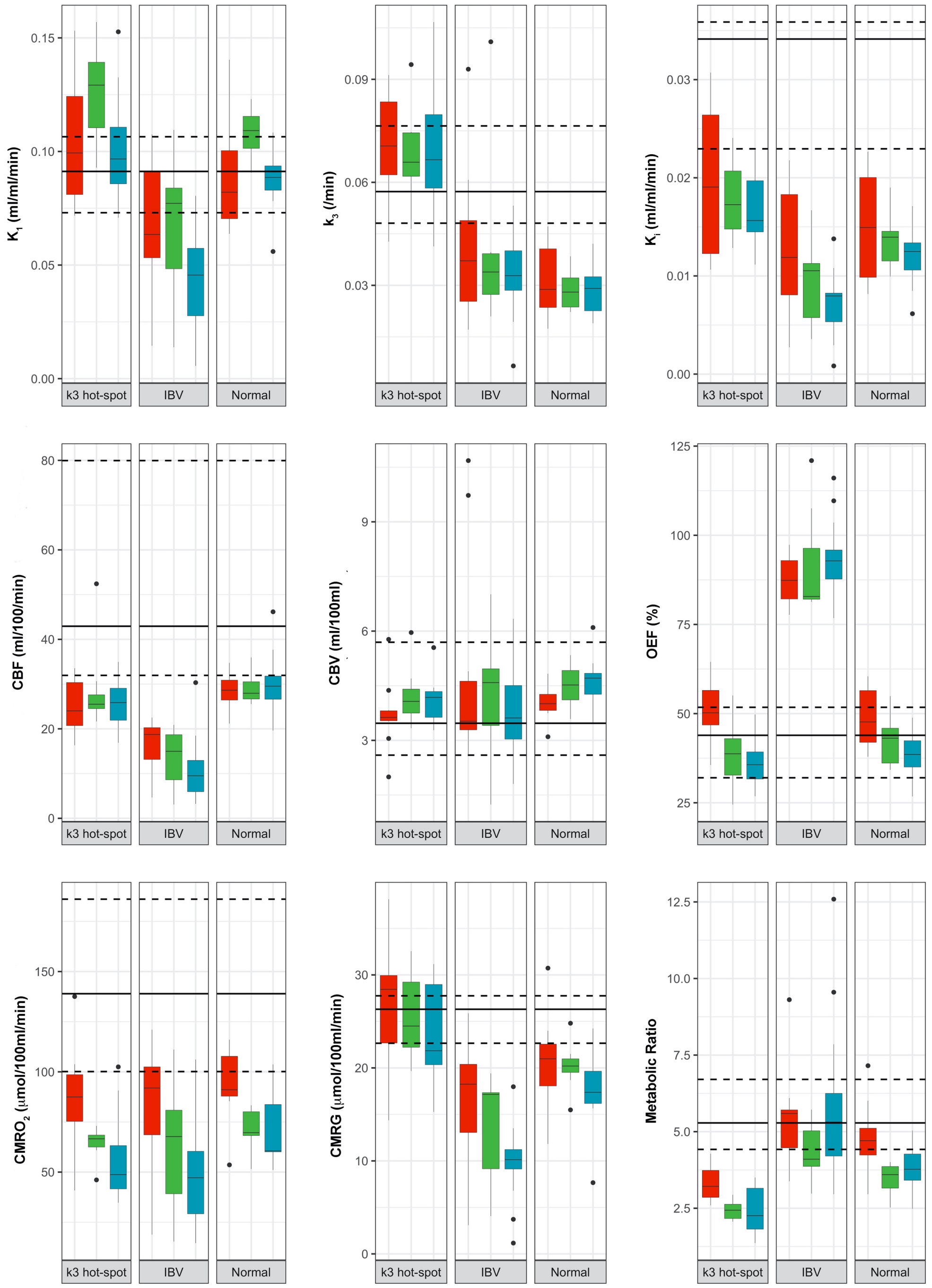

Supplementary Figure 13. Spatial and temporal pattern of metabolic parameters within physiological regions of interest

Box and whisker plots of the kinetic [18]FDG parameters (K_1, k_3, K_i), cerebral blood flow (CBF), blood volume (CBV), oxygen extraction fraction (OEF), oxygen metabolism ($CMRO_2$), cerebral glucose metabolism (CMRG), and $CMRO_2$/CMRG metabolic ratio for the k_3 hot-spot, and ischemic brain volume (IBV) and normal-appearing brain (Normal) regions of interest (ROIs) in patients within 24 hours (Early; red), days 2 – 5 (Intermediate; green) and days 6 – 12 (Late, blue) post injury. The horizontal line within each box denotes the median value, the lower and upper boundaries the 25th and 75th centile, the vertical lines the 10th and 90th centile, and the closed circles outlying data points. The solid and dashed black lines represent the median and the full range of values for healthy volunteers, respectively. For the metabolic ratio the median and range in healthy volunteers is calculated from two different cohorts of subjects.

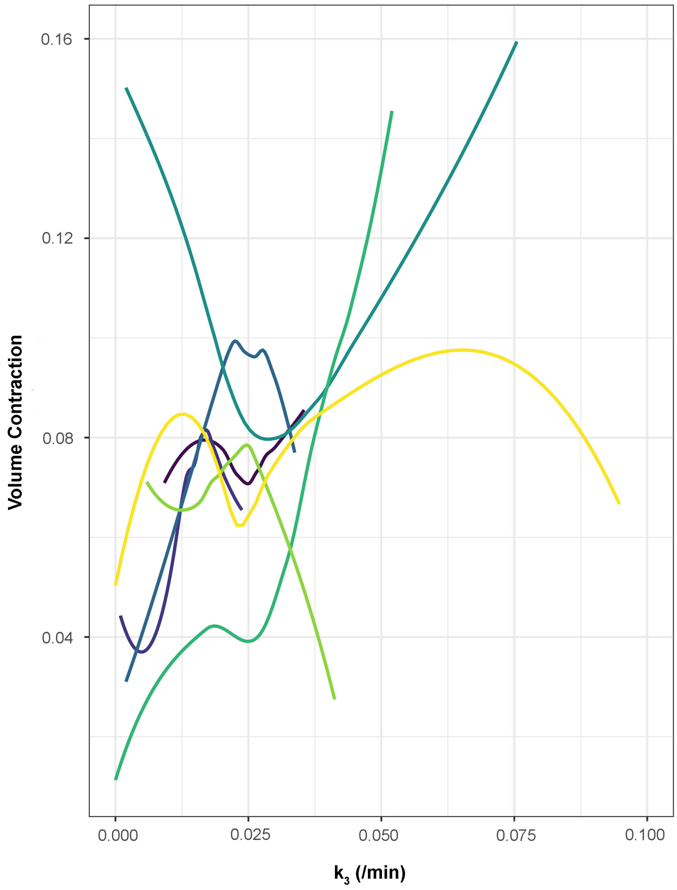

Supplementary Figure 14. Comparison of changes in volumetric structural MRI obtained at follow-up with acute ^{18}F-FDG phosphorylation activity using voxel data from individual patients

For the seven patients with follow-up T1-weighted MRI and early ^{18}F-FDG PET the Jacobian determinant image was used to compare volume contraction/loss within the structural image with ^{18}F-FDG phosphorylation rate (k_3). The fitted relationship is plotted for voxels across the normal appearing brain in individual subjects using locally weighted scatterplot smoothing.

Region	Core				Penumbra				Peri-penumbra				Normal appearing			
**Time point**	**Early**	**Inter**	**Late**	**p-value[A]**	**Early**	**Inter**	**Late**	**p-value[A]**	**Early**	**Inter**	**Late**	**p-value[A]**	**Early**	**Inter**	**Late**	**p-value[A]**
**K_1, ml/ml/min**	0.033 (0.016)	0.048 (0.023)	0.038 (0.021)	0.559	0.046 (0.026)	0.057 (0.015)	0.048 (0.016)	0.382	0.075 (0.015)	0.095 (0.007)	0.084 (0.017)	0.015	0.082 (0.030)	0.109 (0.014)	0.089 (0.011)	0.012
**k_2, /min**	0.122 (0.060)	0.166 (0.042)	0.158 (0.070)	0.344	0.110 (0.066)	0.135 (0.032)	0.133 (0.012)	0.492	0.125 (0.038)[B]	0.185 (0.030)	0.158 (0.043)	0.004	0.152 (0.04)	0.207 (0.043)	0.174 (0.018)	0.017
**k_3, /min**	0.038 (0.010)	0.042 (0.016)	0.055 (0.018)	0.087	0.039 (0.014)	0.034 (0.009)	0.040 (0.006)	0.276	0.030 (0.015)	0.028 (0.008)	0.032 (0.008)	0.879	0.029 (0.017)	0.028 (0.008)	0.029 (0.01)	0.880
**K_i, ml/ml/min**	0.010 (0.006)	0.009 (0.005)	0.010 (0.006)	0.860	0.012 (0.009)	0.012 (0.005)	0.011 (0.003)	0.847	0.016 (0.009)	0.013 (0.003)	0.014 (0.003)	0.765	0.015 (0.010)	0.014 (0.003)	0.012 (0.002)	0.418
**CMRG, μmol/100ml/min**	11.6 (2.8)	12.2 (7.6)	12.1 (6.9)	0.797	17.0 (3.1)	14.8 (3.4)	13.7 (2.7)	0.467	19.9 (4.4)	19.4 (2.2)	18.8 (3.8)	0.470	21.0 (4.5)	20.2 (1.5)	17.4 (3.4)	0.186
**LC**	0.49 (0.03)	0.46 (0.03)	0.50 (0.07)	0.085	0.47 (0.08)	0.45 (0.03)	0.48 (0.03)	0.182	0.47 (0.07)	0.41 (0.02)	0.44 (0.04)	0.031	0.44 (0.06)	0.39 (0.02)	0.41 (0.04)	0.037
**CBF, ml/100ml/min**	9.9 (4.9)	17.9 (6.4)	12.2 (9.8)	0.145	16.6 (3.8)	22.4 (3.1)	17.3 (6.2)	0.051	25.0 (4.6)	30.6 (9.0)	27.4 (8.2)	0.191	28.6 (4.5)	28.0 (3.8)	29.5 (5.2)	0.889
**CBV, ml/100ml**	2.4 (0.8)	3.0 (0.7)	2.7 (0.7)	0.147	2.9 (0.7)	3.2 (1.1)	2.8 (0.4)	0.540	3.4 (0.2)	3.8 (0.8)	3.7 (1.0)	0.523	4.0 (0.5)	4.5 (0.8)	4.7 (0.5)	0.091
**$CMRO_2$, μmol/100ml/min**	32.8 (8.9)	42.4 (26.9)	21.7 (12.6)	0.117	55.5 (12.3)	49.6 (14.6)	37.8 (10.5)	0.022	88.5 (16.8)	68.3 (9.3)	61.9 (19)	0.022	91.1 (19.8)	69.7 (11.9)	60.6 (23.6)	0.003
**OEF, %**	40.9 (19.1)	31.2 (19.8)	24.7 (7.1)	0.017	45.5 (11.6)	30.8 (12.9)	32.2 (12.3)	0.004	49.6 (18)	43.7 (9.4)	38.4 (12.3)	0.028	47.7 (14.5)	43.1 (9.8)	38.6 (7.4)	0.024
**MR**	3.0 (0.9)	2.7 (1.1)	2.1 (0.9)	0.014	3.5 (0.3)	2.9 (0.9)	2.8 (1.3)	0.044	4.4 (0.5)	3.7 (0.8)	3.4 (1.4)	0.060	4.7 (0.9)	3.6 (0.7)	3.8(0.9)	0.008

Supplementary Table 1. Temporal pattern of regional metabolic parameters

^{18}F-FDG kinetic parameters, cerebral glucose metabolism (CMRG), lumped constant (LC), cerebral blood flow (CBF), blood volume (CBV), oxygen metabolism ($CMRO_2$), oxygen extraction fraction (OEF) and $CMRO_2$/CMRG metabolic ratio (MR) for the different regions of interest (ROIs) in patients. Values are median (interquartile range). [A] Kruskal-Wallis test for comparison between early, intermediate and late time points post injury within core, penumbra, peri-penumbra and normal-appearing brain regions in patients, with subsequent post hoc Dunn's tests surviving correction for multiple comparisons ($p < 0.005$) between the early and intermediate time points[B]

	Patients					Healthy volunteers	
	Core	Penumbra	Peri-penumbra	Normal appearing	p-value[A]		p-value[B]
**K_1, ml/ml/min**	0.040 (0.027)[C,D]	0.054 (0.025)[C]	0.083 (0.032)	0.092 (0.039)	<0.001	0.098 (0.023)	0.654
**k_2, /min**	0.183 (0.159)	0.156 (0.096)	0.183 (0.095)	0.197 (0.118)	0.305	0.183 (0.069)	0.2443
**k_3, /min**	0.059 (0.038)[C]	0.057 (0.032)	0.050 (0.024)	0.048 (0.018)	0.003	0.123 (0.044)	<0.001
**k_4, /min**	0.009 (0.011)	0.011 (0.007)	0.012 (0.009)	0.013 (0.007)	0.469	0.012 (0.004)	0.976
**K_i, ml/ml/min**	0.012 (0.006)[C,D]	0.016 (0.005)	0.019 (0.006)	0.017 (0.005)	<0.001	0.040 (0.012)	<0.001

Supplementary Table 2. ^{18}F-FDG kinetic parameters from regional kinetic modelling

^{18}F-FDG kinetic parameters (K_1, k_2, k_3, k_4, and K_i) for the different regions of interest (ROIs) in patients and healthy volunteers. Values are median (interquartile range). [A] Kruskal-Wallis test for comparison between core, penumbra, peri-penumbra and normal-appearing ROIs within patients, with subsequent post hoc Dunn's tests surviving correction for multiple comparisons ($p < 0.005$) between each lesion ROI and normal-appearing brain[C], and between core and peri-penumbra[D]. [B] Mann-Whitney U test for comparison between the normal-appearing ROI in patients versus healthy volunteers.

	Patients			Healthy volunteers	
	k_3 hot-spot	IBV	Normal		p-value[A]
K_1, ml/ml/min	0.101 (0.032)[B]	0.055 (0.040)[E]	0.091 (0.025)	0.091 (0.007)	<0.001
k_2, /min	0.326 (0.120)[B,C,D]	0.174 (0.067)	0.173 (0.055)	0.128 (0.029)	<0.001
k_3, /min	0.067 (0.020)[B,C]	0.034 (0.017)	0.029 (0.010)	0.057 (0.009)	<0.001
K_i, ml/ml/min	0.018 (0.006)[B]	0.008 (0.006)[F]	0.013 (0.005)	0.034 (0.008)	<0.001
CMRG, μmol/100ml/min	24.1 (9.1)[B,C]	12.8 (8.4)[E,F]	19.9 (4.9)	26.3 (1.9)	<0.001
LC	0.43 (0.03)[D]	0.44 (0.03)[F]	0.41 (0.04)	0.56 (0.04)	<0.001
CBF, ml/100 ml/min	25.0 (7.6)[B,D]	13.7 (12.3)[E,F]	28.6 (4.9)	42.9 (11.5)	<0.001
CBV, ml/100 ml	3.9 (0.8)	3.7 (1.7)	4.3 (0.9)	3.5 (0.5)	<0.001
$CMRO_2$, μmol/100ml/min	67.3 (39.1)[D]	64.7 (58.5)[F]	82.1 (30.5)	139.0 (39.0)	<0.001
OEF, %	41.1 (15.6)[B]	88.8 (13.0)[E,F]	42.1 (10.4)	43.9 (4.5)	<0.001
MR	2.7 (1.1)[B,C]	5.2 (1.8)	4.0 (1.2)		<0.001
Volume (ml)	64.0 (40.8)	6.8 (16.9)[G]			

Supplementary Table 3. Comparison of physiology within ^{18}F-FDG phosphorylation hot-spots, the ischaemic brain volume, normal-appearing brain and healthy volunteers

^{18}F-FDG kinetic parameters, cerebral glucose metabolism (CMRG), lumped constant (LC), cerebral blood flow (CBF), blood volume (CBV), oxygen metabolism ($CMRO_2$), oxygen extraction fraction (OEF) and $CMRO_2$/CMRG metabolic ratio (MR) for patient regions with an increase in ^{18}F-FDG phosphorylation (k_3 hot-spot), the ischaemic brain volume (IBV) and normal-appearing brain (Normal), and healthy volunteers. Values are median (interquartile range). [A] Kruskal-Wallis test for comparisons between the different groups, with subsequent post hoc Dunn's tests surviving correction for multiple comparisons ($p < 0.005$) between k_3 hot-spots and IBV[B], normal-appearing brain in patients[C] and healthy volunteers[D], and between IBV and normal-appearing brain in patients[E] and healthy volunteers[F]. [G]Mann-Whitney U test for the comparison between volume of k_3 hot-spot and IBV in patients ($p < 0.001$).

Region	k_3 hot-spot				IBV			
Time point	Early	Inter	Late	p-value[A]	Early	Inter	Late	p-value[A]
K_1, ml/ml/min	0.099 (0.043)	0.129 (0.029)	0.097 (0.025)	0.066	0.063 (0.038)	0.077 (0.036)	0.0456 (0.030)	0.056
k_2, /min	0.297 (0.058)	0.404 (0.124)	0.334 (0.122)	0.058	0.160 (0.044)	0.192 (0.074)	0.178 (0.061)	0.574
k_3, /min	0.071 (0.021)	0.066 (0.013)	0.067 (0.021)	0.888	0.037 (0.024)	0.034 (0.012)	0.033 (0.012)	0.946
K_i, ml/ml/min	0.019 (0.014)	0.017 (0.006)	0.016 (0.005)	0.584	0.012 (0.010)	0.011 (0.006)	0.008 (0.003)	0.087
CMRG, µmol/100ml/min	28.4 (7.3)	24.5 (7.0)	21.8 (8.6)	0.265	18.2 (7.3)	17.2 (8.2)	10.1 (2.1)	0.046
LC	0.45 (0.03)	0.41 (0.03)	0.43 (0.03)	0.058	0.44 (0.05)	0.42 (0.07)	0.44 (0.02)	0.440
CBF, ml/100ml/min	24.0 (9.6)	25.5 (3.1)	25.9 (7.2)	0.678	18.7 (7.1)	15.0 (10.1)	9.5 (7.0)	0.106
CBV, ml/100ml	3.6 (0.3)	4.1 (0.7)	4.2 (0.7)	0.284	3.5 (1.3)	4.6 (1.6)	3.6 (1.5)	0.842
$CMRO_2$, µmol/100ml/min	87.5 (23.3)	66.6 (6.0)	48.7 (21.6)	0.007	91.9 (33.9)	67.7 (41.7)	47.2 (31.2)	0.058
OEF, %	50.2 (9.7)	38.7 (10.2)	35.7 (7.6)	0.002	87.4 (10.8)	82.8 (14.3)	92.8 (8.1)	0.277
MR	3.2 (0.9)	2.4 (0.5)	2.3 (1.3)	0.006	5.6 (2.0)	4.1 (1.2)	5.3 (2.0)	0.319
Volume (ml)	63.4 (41.8)	69.6 (21.3)	62.9 (92.4)	0.838	7.8 (32.5)	9.7 (8.7)	6.0 (9.6)	0.771

Supplementary Table 4. Temporal pattern of metabolic parameters within physiological regions

^{18}F-FDG kinetic parameters, cerebral glucose metabolism (CMRG), lumped constant (LC), cerebral blood flow (CBF), blood volume (CBV), oxygen metabolism ($CMRO_2$), oxygen extraction fraction (OEF) and $CMRO_2$/CMRG metabolic ratio (MR) for the k_3 hot-spot and ischemic brain volume (IBV). Values are median (interquartile range). [A] Kruskal-Wallis test for comparison between early, intermediate and late time points post injury within k_3 hot-spot and IBV regions.

References

1. Kinahan PE, Rogers JG. Analytic 3D image reconstruction using all detected events. *IEEE Trans Nucl Sci.* 1989;36:964-968.
2. Frackowiak RS, Lenzi GL, Jones T, Heather JD. Quantitative measurement of regional cerebral blood flow and oxygen metabolism in man using 15O and positron emission tomography: theory, procedure, and normal values. *J Comput Assist Tomogr.* 1980;4(6):727-736.
3. Lammertsma AA, Baron JC, Jones T. Correction for intravascular activity in the oxygen-15 steady-state technique is independent of the regional hematocrit. *J Cereb Blood Flow Metab.* 1987;7(3):372-374.
4. Herscovitch P, Raichle ME. What is the correct value for the brain-blood partition coefficient for water? *J Cereb Blood Flow Metab.* 1985;5(1):65-69.
5. Phelps ME, Huang SC, Hoffman EJ, Kuhl DE. Validation of tomographic measurement of cerebral blood volume with C-11-labeled carboxyhemoglobin. *J Nucl Med.* 1979;20(4):328-334.
6. Hong YT, Fryer TD. Kinetic modelling using basis functions derived from two-tissue compartmental models with a plasma input function: general principle and application to [18F]fluorodeoxyglucose positron emission tomography. *Neuroimage.* 2010;51(1):164-172.
7. Sokoloff L, Reivich M, Kennedy C, Des Rosiers MH, Patlak CS, Pettigrew KD*, et al.* The [^{14}C]deoxyglucose method for the measurement of local cerebral glucose utilization: theory, procedure, and normal values in the conscious and anesthetized albino rat. Journal of neurochemistry 1977; 28(5): 897-916.
8. Kuwabara H, Evans AC, Gjedde A. Michaelis-Menten constraints improved cerebral glucose metabolism and regional lumped constant measurements with [18F]fluorodeoxyglucose. *J Cereb Blood Flow Metab.* 1990;10(2):180-189.
9. Gjedde A and Diemer NH. Autoradiographic Determination of Regional Brain Glucose Content. *J Cereb Blood Flow Metab.*1983;3:303-310.
10. Coles JP, Fryer TD, Smielewski P, et al. Defining ischemic burden after traumatic brain injury using 15O PET imaging of cerebral physiology. *J Cereb Blood Flow Metab.* 2004;24(2):191-201.
11. Coles JP, Fryer TD, Smielewski P, et al. Incidence and mechanisms of cerebral ischemia in early clinical head injury. *J Cereb Blood Flow Metab.* 2004;24(2):202-211.
12. Powers WJ, Grubb RL, Jr., Darriet D, Raichle ME. Cerebral blood flow and cerebral metabolic rate of oxygen requirements for cerebral function and viability in humans. *J Cereb Blood Flow Metab.* 1985;5(4):600-608.
13. Yundt KD, Diringer MN. The use of hyperventilation and its impact on cerebral ischemia in the treatment of traumatic brain injury. *Crit Care Clin.* 1997;13(1):163-184.
14. Avants BB, Epstein CL, Grossman M, Gee JC. Symmetric diffeomorphic image registration with cross-correlation: evaluating automated labeling of elderly and neurodegenerative brain. *Med Image Anal* 2008;12(1):26-41.
15. Ledig C, Heckemann RA, Hammers A*,* et al. Robust whole-brain segmentation: application to traumatic brain injury. *Med Image Anal* 2015; 21(1): 40-58.
16. Taylor JR, Williams N, Cusack R, et al. The Cambridge Centre for Ageing and Neuroscience (CamCAN) data repository: Structural and functional MRI, MEG, and cognitive data from a cross-sectional adult lifespan sample. *NeuroImage* 2016. doi: 10.1016/j.neuroimage.2015.09.018.
17. Shafto MA, Tyler LK, Dixon M, et al. The Cambridge Centre for Ageing and Neuroscience (CamCAN) study protocol: a cross-sectional, lifespan, multidisciplinary examination of healthy cognitive ageing. *BMC Neurology* 2014; 14(204). doi:10.1186/s12883-014-0204-1.

18. Sanchez-Guerrero A, Mur-Bonet G, Vidal-Jorge M, et al. Reappraisal of the reference levels for energy metabolites in the extracellular fluid of the human brain. *J Cereb Blood Flow Metab.* 2017;37(8):2742-2755.
